# Integrative *in silico* and biochemical analyses demonstrate direct Arl3-mediated ODA16 release from the intraflagellar transport machinery

**DOI:** 10.1016/j.jbc.2025.108237

**Published:** 2025-01-27

**Authors:** Jiaolong Wang, Rune T. Kidmose, Niels Boegholm, Nevin K. Zacharia, Mads B. Thomsen, Anni Christensen, Tara Malik, Karl Lechtreck, Esben Lorentzen

**Affiliations:** 1 Department of Molecular Biology and Genetics, Aarhus University, Aarhus C, Denmark; 2 Department of Cellular Biology, University of Georgia, Athens, Georgia, USA

**Keywords:** cilium, flagellum, intraflagellar transport, outer dynein arms, IFT46, ODA16, Arl3, IDA3, ODA8, FAP20

## Abstract

Outer dynein arms (ODAs) are essential for ciliary motility and are preassembled in the cytoplasm before trafficking into cilia by intraflagellar transport (IFT). ODA16 is a key adaptor protein that links ODAs to the IFT machinery *via* direct interaction with the IFT46 protein. However, the molecular mechanisms regulating the assembly, transport, and release of ODAs remain poorly understood. Here, we employ AlphaPulldown, an *in silico* screening method, to identify direct interactors of ODA16, including the dynein adaptor IDA3 and the small GTPase Arl3. We use structural modeling, biochemical, and biophysical assays on *Chlamydomonas* and human proteins to elucidate the interactions and regulatory mechanisms governing the IFT of ODAs. We identify a conserved N-terminal motif in *Chlamydomonas* IFT46 that mediates its binding to one side of the ODA16 structure. Biochemical dissection reveals that IDA3 and Arl3 bind to the same surface of ODA16 (the C-terminal **β**-propeller face), which is opposite to the IFT46 binding site, enabling them to dissociate ODA16 from IFT46, likely through an allosteric mechanism. Our findings provide mechanistic insights into the concerted actions of IFT and adaptor proteins in ODA transport and regulation.

Motile cilia and flagella are essential organelles that power the movement of cells and extracellular fluids, playing crucial roles in various physiological processes, such as mucus clearance, cerebrospinal fluid flow, and left-right body patterning (1). The beating of these organelles relies on the coordinated activity of multiple dynein motors, including the outer dynein arms (ODAs) and inner dynein arms (IDAs) (2). ODAs are large, multi-subunit complexes that generate the force for ciliary beating by hydrolyzing ATP and inducing microtubule sliding (3). In the green algae *Chlamydomonas reinhardtii* (Cr), ODAs are composed of >15 subunits including three heavy chains (HC), two intermediate chains, and >10 light chains (3).

The assembly of ODAs occurs in the cytoplasm with the help of assembly factors, and the resulting complexes are then transported into cilia by intraflagellar transport (IFT) and docked at the axoneme *via* the ODA docking complex (ODA-DC) (4, 5, 6, 7, 8). Recent work has shown that the assembly factor Shulin packages ODAs into a closed conformation during this transport phase (9). IFT is a bidirectional transport system that moves ciliary precursors and turnover products between the cell body and the ciliary tip (10, 11). The IFT machinery consists of large protein complexes called IFT trains that are composed of IFT-A and IFT-B subcomplexes and are driven by kinesin and IFT dynein motors (12, 13).

Previous studies have identified ODA16 as a key adaptor protein that links ODAs to the IFT machinery. In *Chlamydomonas*, ODA16 directly interacts with the IFT-B subunit IFT46 and is required for the transport of ODAs into flagella (14, 15, 16). The crystal structure of ODA16 revealed that it consists of two domains: a small N-terminal domain and a C-terminal β-propeller (17). The N-terminal region of IFT46 likely binds to a cleft between these two domains of ODA16, while the C-terminal β-propeller of ODA16 is necessary for its association with ODAs (17). Thus, ODA16 functions as a cargo adaptor, with its C-terminus binding ODAs and its N-terminus associating with IFT46 to facilitate transport (reviewed in (18)). This dual-binding capability allows ODA16 to effectively link ODAs to the IFT machinery for ciliary transport. Interestingly, human IFT46 does not appear to interact directly with human ODA16 suggesting species-specific mechanisms for ODA trafficking (19).

Another important adaptor protein is IDA3, which is required for the IFT of the double-headed inner dynein arm I1/f in *Chlamydomonas* (6, 20, 21). IDA3 moves by anterograde IFT to the ciliary tip, and its transport frequency is highly upregulated during ciliary growth (20). IDA3 and the IC140 subunit of IDA I1/f can be co-immunoprecipitated from ciliary extracts suggesting an interaction (20). Interestingly, IDA3 continues to move by IFT even in the absence of IDA I1/f, revealing cargo-independent binding to IFT trains (20).

The small GTPase Arl3 has been implicated in the regulation of ciliary protein trafficking (22, 23). Arl3 plays a crucial role in releasing lipidated cargo proteins, such as the myristoylated ciliary protein UNC119, in the cilium (22). UNC119 binds to and shuttles myristoylated proteins, including G protein α-subunits and nephrocystin-3, into the cilium (24). Upon entering the cilium, Arl3 in its active GTP-bound state interacts with UNC119, causing a conformational change that leads to the release of the myristoylated cargo (22, 24). The activation of Arl3 is regulated by the guanine nucleotide exchange factor (GEF) Arl13b, another small GTPase that is specifically localized to the cilium (25). Arl13b interacts with Arl3 and promotes the exchange of GDP for GTP, thereby activating Arl3. Mutations in Arl3 and Arl13b cause Joubert syndrome, a ciliopathy characterized by cerebellar abnormalities, retinal dystrophy, and nephronophthisis (26). The loss of Arl13b function leads to impaired Arl3 activation and subsequent defects in the release of lipidated cargo proteins within the cilium, highlighting the importance of this regulatory pathway in ciliary protein trafficking and function (25, 26).

A recent paper from Huang and colleagues provides important insight into how Arl3 in *Trypanosoma brucei* (Tb) facilitates the unloading of ODA16 from IFT trains in motile cilia (27). The study found that in the absence of TbArl3A and TbArl3C, TbODA16 accumulated in the cilia, and its interaction with IFT components, such as TbIFT88, TbIFT46, TbIFT20, and TbIFT81, was stabilized. Importantly, active TbArl3A and TbArl3C variants were able to displace TbODA16 from the IFT complex. These findings suggest a model where active Arl3 binds to ODA16 and dissociates it from the IFT machinery. The study also showed that TbArl13b depletion led to TbODA16 accumulation in the cilia (27). These observations could be reproduced in human cells (27), although the biochemical mechanism of ODA16 release from IFT trains by Arl3 remains to be elucidated.

Despite significant advances in our understanding of ciliary protein trafficking, several key questions remain unanswered regarding the precise mechanisms of ODA assembly, transport, and regulation. In this study, we sought to address the following critical questions (a): How does ODA16 interact with the IFT machinery, and are these interactions conserved across species? (b) What are the direct protein-protein interactions involved in ODA trafficking, and how are they regulated? (c) What is the molecular basis for the release of ODA16 from IFT trains, and how might this process be controlled? (d) The combination of *in silico* structural modeling with biochemical and biophysical approaches provides a comprehensive molecular framework for understanding the intricate processes governing ODA trafficking.

## Results

### A conserved N-terminal CrIFT46 motif mediates the interaction with CrODA16

**Figure 1:**
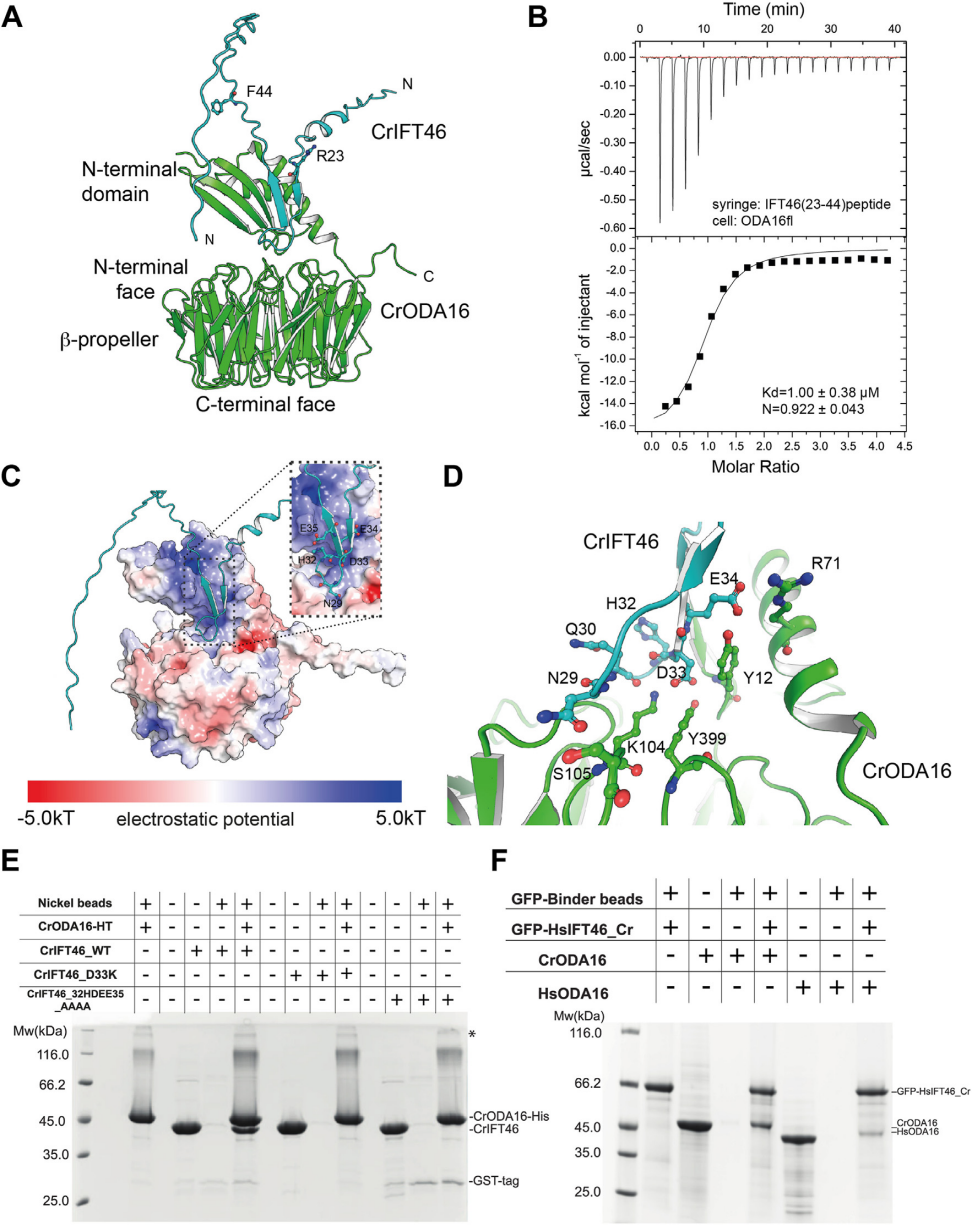
**A conserved N-terminal motif in CrIFT46 binds CrODA16**. *A*, alphaFold-predicted structural model of the CrODA16-IFT46 complex. CrODA16 (*green*) consists of an N-terminal domain and a C-terminal β-propeller. CrIFT46 (*cyan*) binds in a cleft between these domains. R23 and F44 of CrIFT46, shown as sticks, mark the N- and C-termini of the peptide used in panel *B*. *B*, isothermal titration calorimetry (ITC) of the CrIFT46(23-44) peptide with CrODA16fl demonstrates binding with a Kd of 1.00 ± 0.38 μM. *C*, electrostatic surface potential of CrODA16 reveals a positively charged binding pocket (*blue*) that accommodates the negatively charged 32HDEE35 motif of CrIFT46 (*inset*). *D*, close-up view of the CrODA16-CrIFT46 interface, highlighting key residues involved in polar interactions. *E*, pull-down assay with immobilized CrODA16-HT (his-tagged) shows that both CrIFT46 mutants (D33K and 32HDEE35_AAAA) disrupt the interaction with CrODA16. The *asterisk* indicates impurities. *F*, pull-down assay demonstrating that a chimeric HsIFT46 construct (HsIFT46_Cr), where residues 4–26 are replaced with CrIFT46 residues 22–44, interacts with both HsODA16 and CrODA16. GFP-tagged HsIFT46_Cr was immobilized on GFP-Binder beads.

In *Chlamydomonas*, ODAs are transported into cilia *via* the adaptor protein ODA16, which associates with the IFT machinery through recognition of the N-terminal 147 amino acids of IFT46 (14, 15, 16, 17, 28). The crystal structure of *Chlamydomonas* ODA16 (CrODA16) demonstrated a small N-terminal domain (residues 1–80) situated on top of the C-terminal β-propeller (residues 80–445), and biochemical experiments showed that both CrODA16 domains are required for interaction with *Chlamydomonas reinhartii* IFT46 (CrIFT46) (17). However, there is no experimental structure to elucidate how CrODA16 docks onto IFT trains *via* IFT46 (17). To address this, we used AlphaFold-Multimer (29) and Alphafold 3 (30) to predict the structure of the CrIFT46-ODA16 complex (Fig. 1*A*). The structural model of CrODA16-IFT46 has excellent confidence scores (for predicted local distance difference test (pLDDT) scores and predicted aligned error (PAE) plot, see Fig. S1*A*) and suggests that residues 23 to 44 of CrIFT46 complement the β-sheet of the CrODA16 N-terminal domain while the preceding loop of CrIFT46 makes side-chain-specific interactions with β-propeller residues of CrODA16 (Fig. 1*A*). Interestingly, this mode of interaction agrees well with the previous biochemical observation that both the N- and the C-terminal domains of CrODA16 are required for association with CrIFT46 (17). To validate the structural prediction that CrIFT46_23-44_ constitutes the CrODA16 binding fragment, this peptide was synthesized and used to titrate CrODA16 in isothermal titration calorimetry (ITC) experiments (Fig. 1*B*). The results show that CrIFT46_23-44_ binds CrODA16 with a dissociation constant (Kd) of 1.00 ± 0.38 μM, which is comparable to the Kd of 200 ± 72 nM measured in ITC using full-length proteins (17). We conclude that residues 23 to 44 of CrIFT46 constitute the main binding region toward CrODA16.

Mapping of the electrostatic surface potential onto the structure of CrODA16 reveals that the cleft formed between the two domains of CrODA16 is positively charged (Fig. 1*C*). This positively charged cleft in CrODA16 is complemented by the 29-Nxxx(D/E)E−34 motif of the CrIFT46₂₃₋₄₄ fragment, which is predicted to engage in multiple salt bridges and hydrogen bonds with residues from both CrODA16 domains (Fig. 1*D*). We note that the hydrophilic interface agrees with the salt-labile nature of the CrODA16-IFT46 complex reported previously (17). As Asp33 of CrIFT46 is central to the interaction with CrODA16 (Fig. 1*D*), we mutated this residue to a lysine and performed a pull-down experiment of wild-type (WT) or D33K CrIFT46 with hexa-His-tagged (HT) CrODA16. The result shows that whereas WT CrIFT46 is efficiently pulled down by CrODA16-HT, the single D33K point mutation is sufficient to abolish the interaction (Fig. 1*E*). Additionally, complete alanine substitution of the CrIFT46 32-HDEE-35 motif (CrIFT46_32HDEE35_AAAA) also abolishes the interaction with ODA16 in pull-downs (Fig. 1*E*). These results validate the structural model and demonstrate the importance of the CrIFT46 29-Nxxx(D/E)E−34 motif in mediating the association with CrODA16.

To investigate if the ODA16-binding Nxxx(D/E)E motif is conserved in IFT46 of other ciliated organisms, a sequence alignment of the N-terminal part of IFT46 was carried out (Fig. S1*B*). The alignment shows that the motif is conserved in *T. brucei*, *Danio rerio* (Dr)*, Tetrahymena thermophila* (Tt), *Xenopus laevis* (Xl), and *Caenorhabditis elegans* (Ce), suggesting that the IFT46/ODA16 complex formation described here for *Chlamydomonas* is conserved in these organisms. However, the Nxxx(D/E)E motif is not conserved in the mammalian IFT46 sequences from *Homo sapiens* (Hs), *Mus musculus* (Mm), or *Bos taurus* (Bt) (Fig. S1*B*), an observation that is consistent with the fact that HsODA16 does not bind directly to HsIFT46 (19). Interestingly, when examining the cleft in HsODA16, we note that the binding site for the CrIFT46 Nxxx(D/E)E motif is largely conserved (Fig. S1, *C* and *D*). We thus engineered a chimera IFT46 protein where HsIFT46 has residues 4 to 26 replaced with residues 22 to 44 from CrIFT46 containing the Nxxx(D/E)E motif (we name this chimera protein HsIFT46_Cr). Intriguingly, the HsIFT46_Cr protein shows binding to both HsODA16 and CrODA16 (Figs. 1*F* and S1*E*). We conclude that the CrIFT46-binding motif is conserved in human ODA16, although the functional role of this binding site is currently unknown given that HsIFT46 does not have the Nxxx(D/E)E motif conserved. We note that Nxxx(D/E)E is a commonly found motif in human IFT-B proteins, but it is currently not clear if these are responsible for HsODA16 binding. Having established the molecular basis of the CrIFT46-ODA16 interaction, we next sought to identify additional proteins that might interact with ODA16 and regulate its function in ciliary trafficking.

### *In silico* prediction of ODA16 interacting proteins

**Figure 2:**
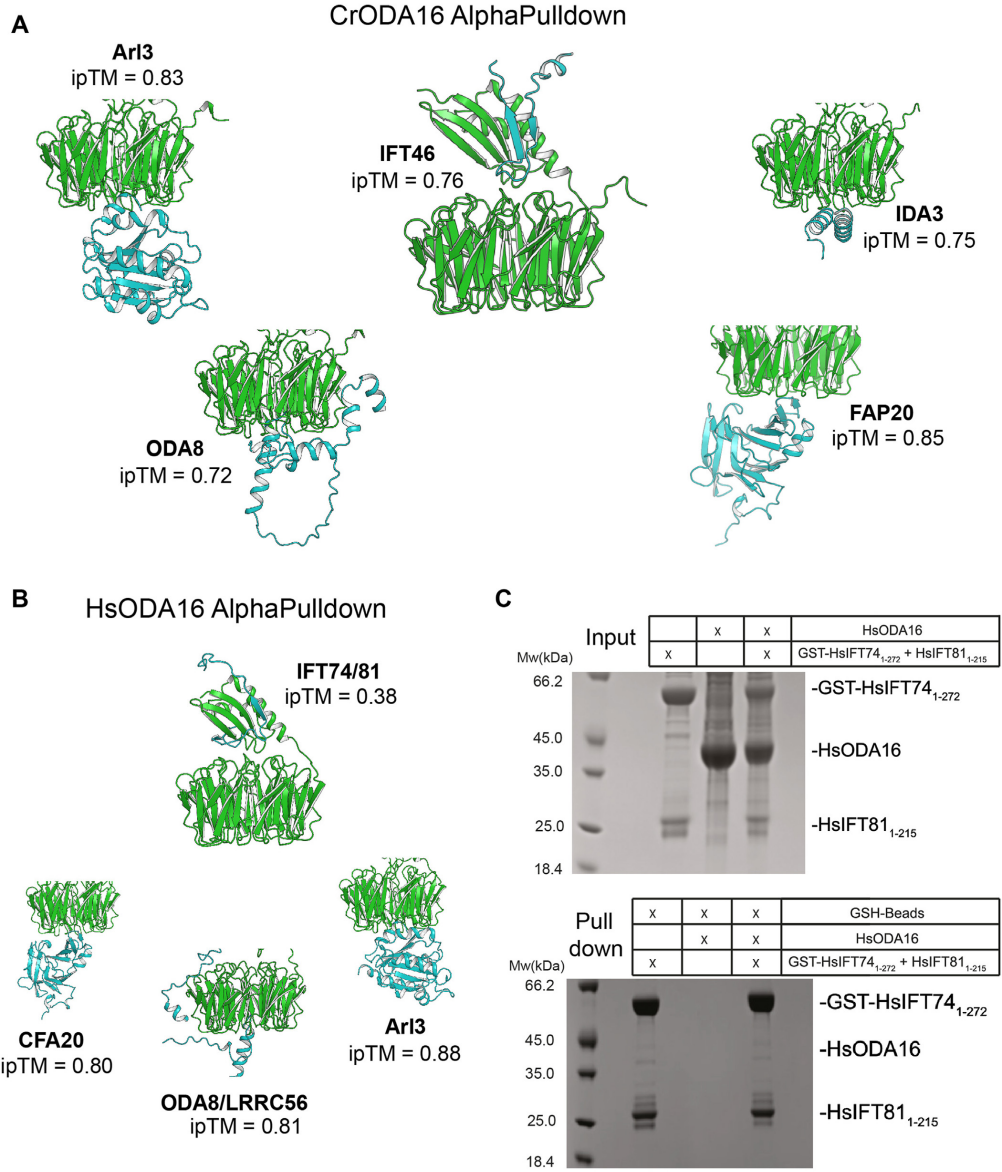
***In silico* prediction of ODA16 interacting proteins using AlphaPulldown.***A*, AlphaPulldown results showing predicted interactors for *Chlamydomonas reinhardtii* (Cr) ODA16. Interacting partners are shown as structural models with CrODA16 (*green*) and the predicted interacting protein (*cyan*). Numbers below each model represent the interface predicted TM-score (ipTM) from AlphaFold 3. ipTM values >0.8 are considered high-quality confident predictions, scores between 0.6–0.8 represent possible interactions and values below 0.6 are considered unlikely interactions. *B*, AlphaPulldown results for *Homo sapiens* (Hs) ODA16, displayed similarly to *panelA*. Note the low ipTM score (0.38) for the HsIFT74/81 interaction, suggesting an unlikely prediction. *C*, experimental investigation of the HsODA16-HsIFT74/81 interaction. *Upper panel*: Input samples showing the presence of GST-HsIFT74_1-272_, HsIFT81_1-215_, and HsODA16. *Lower panel*: Pull-down results using GSH-beads. The absence of HsODA16 in the pull-down fraction (lane 4) indicates no direct interaction between HsODA16 and the HsIFT74_1-272_/81_1-215_ complex, supporting the low ipTM score observed in *panel B*.

ODA16 is known to couple ODAs to the IFT machinery *via* IFT46 (14, 15, 16, 17, 28). Although the C-terminal β-propeller of ODA16 is sufficient for association with ODAs from flagellar extracts (17) and has highly conserved surface areas (19), direct interactors are not known. To this end, we performed *in silico* pull-downs using AlphaPulldown (31) based on the AlphaFold 2 algorithm (32) with CrODA16 or HsODA16 as bait proteins. As preys, we used the top 1000 entries from the database of *Chlamydomonas* or human flagellar proteins carefully curated by Greg Pazour (http://chlamyfp.org/ChlamyFPv2/index.php; (33)). Lists of potentially ODA16 interacting proteins were sorted according to the combined predicted template modeling (pTM) and interface predicted template modeling (ipTM) scores reporting on the confidence of the structure prediction and relative positions of subunits within the putative protein complexes (see Table S1). Hits with a combined pTM/ipTM score above 0.6 were manually passed through the AlphaFold 3 server (30). Structures of ODA16 complexes predicted by AlphaFold 3 with low PAE scores for interacting regions and ipTM>0.7 were selected as high-confidence hits (Fig. 2*A*).

Most high-confidence direct interactors of ODA16 were common to Hs and Cr including ODA8, Arl3, and FAP20 (also known as CFAP20 or BUG22) (Fig. 2, *A* and *B*). These ODA16 interactors are all predicted to associate with the C-terminal face of the ODA16 β-propeller and have robust PAE plots and high-confidence ipTM values (Figs. 2, *A* and *B* and S2). All three proteins have important ciliary functions with ODA8 being an ODA assembly factor (34), Arl3 being a small GTPase important for the ciliary release of lipidated proteins (22), and the FAP20 protein associating with microtubule doublets of the ciliary axoneme (35). More specifically, FAP20 forms a heterodimeric complex with PACRG to tether A- and B tubules together in the microtubule doublets (36). Interestingly, it was previously shown that the mouse *arl13b* mutant (known as hennin or hnn) has disrupted outer doublet organization with the B-tubule often failing to properly attach to the A-tubule (37). Although the ODA16-FAP20 interaction is not further explored here, the data agree with a role for ODA16 in the ciliary trafficking of FAP20. In addition to the three ODA16 interactors common to both Hs and Cr, IDA3, which is *Chlamydomonas* specific adaptor protein for the IFT of IDA I1/f (20, 21, 38), was predicted as a direct interactor of CrODA16 (Fig. 2*A*). IDA3 is also predicted to bind ODA16 at the C-terminal face of the β-propeller (Fig. 2*A*), and the direct ODA16-IDA3 interaction suggests a potential coupling between the ciliary trafficking of outer and inner dynein arms.

The absence of a direct association between HsODA16 and HsIFT46 raises questions about how human ODA16 interfaces with the IFT machinery. To address this, we conducted an AlphaFold 3 screen of HsODA16 against all human IFT-B proteins. This analysis suggested that the unstructured N-terminus of IFT74 within the IFT81/74 subcomplex might interact with ODA16 in a manner analogous to the CrIFT46-CrODA16 interaction (Figs. 2*B* and S2). However, closer examination revealed potential issues with this prediction. Despite reasonably good PAE scores (Fig. S2*B*), the HsIFT74-ODA16 structural model yielded a low ipTM score of 0.38. Furthermore, the pLDDT values for HsIFT74 were notably low (Fig. S2*A*), suggesting this prediction might be a false positive. To further examine this *in silico* prediction, we conducted a pull-down experiment, which failed to demonstrate a direct interaction between HsODA16 and HsIFT74/81 (Fig. 2*C*). Consequently, the mechanism by which human ODA16 couples to the IFT machinery remains elusive, underscoring the need for further investigation into species-specific adaptations in ciliary protein transport.

### CrIDA3 associates directly with the **β**-propeller of CrODA16

**Figure 3:**
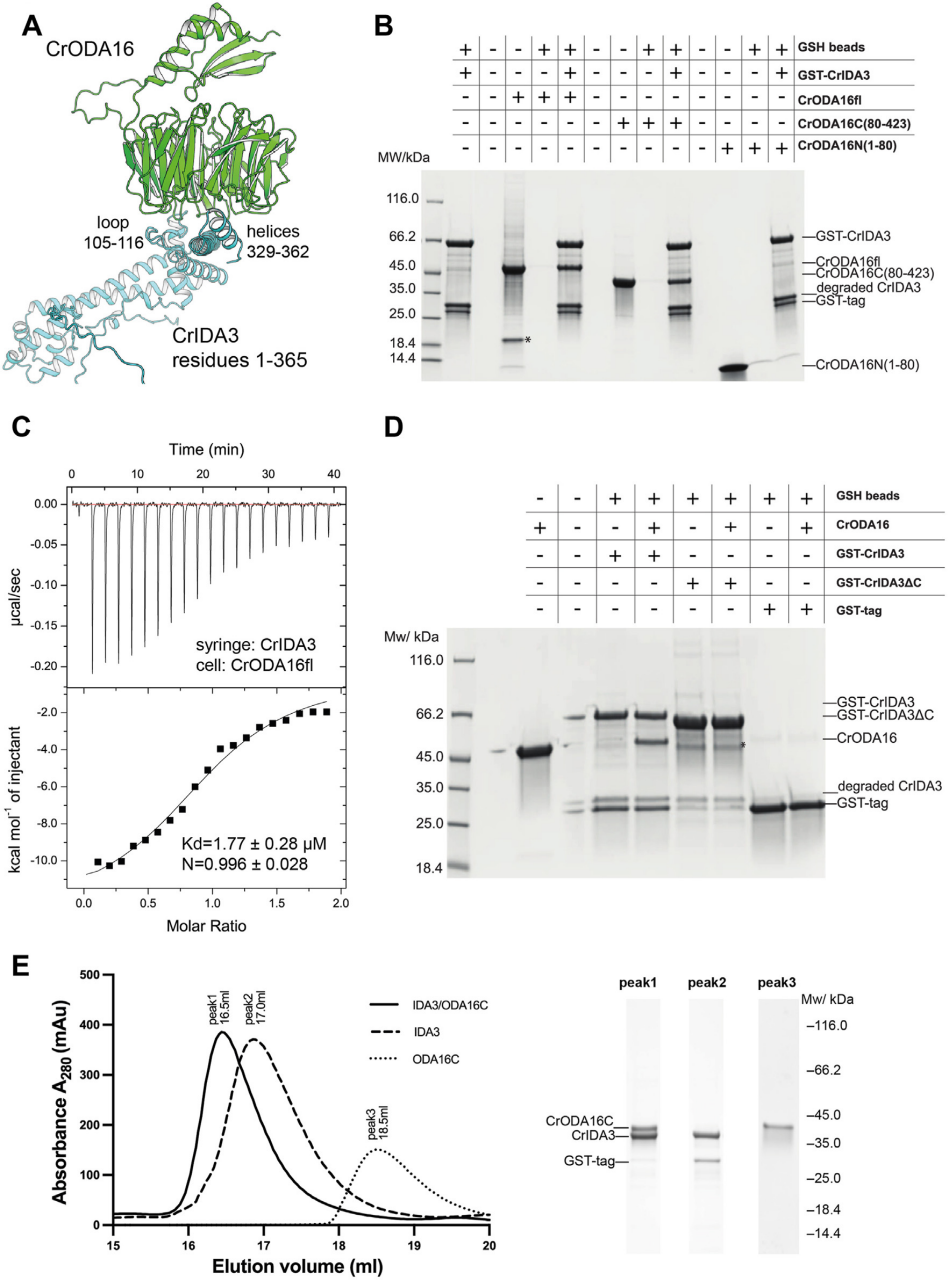
**Characterization of the interaction between CrIDA3 and CrODA16.***A*, structural prediction of CrODA16 (*green*) in complex with CrIDA3 residues 1 to 365 (*cyan*). Key structural elements are labeled. *B*, GSH pull-down assay with GST-tagged CrIDA3 and various CrODA16 constructs. The gel shows that the C-terminal domain of CrODA16 (CrODA16fl and CrODA16C(80–423)) binds to CrIDA3, while the N-terminal domain (CrODA16N(1–80)) does not. *C*, isothermal titration calorimetry (ITC) of CrIDA3 with CrODA16fl. The binding curve indicates a Kd of 1.77 ± 0.28 μM and a stoichiometry (N) of 0.996 ± 0.028. *D*, GSH pull-down assay comparing the binding of CrODA16 to full-length GST-CrIDA3 and GST-CrIDA3ΔC (lacking the C-terminal α-helices: residues 333–366). The gel shows that the C-terminal α-helices of CrIDA3 are required for interaction with CrODA16. The *asterisk* indicates an impuritie. *E*, size exclusion chromatography (SEC; Superose 6 Increase 10/300 column was used throughout this study) profiles (*left panel*) of CrIDA3-ODA16C complex, individual CrIDA3, and CrODA16C. The corresponding SDS-PAGE analysis of SEC peaks (*right panel*) confirms the formation of a stable CrIDA3-ODA16C complex in peak 1.

Among the predicted interactors, we were particularly intrigued by IDA3, given its known role in IDA transport. We therefore investigated the molecular details of its interaction with ODA16. IDA3 is a *Chlamydomonas*-specific adaptor protein required for the IFT of IDA I1/f, and this double heavy chain containing IDA was shown to be specifically missing from cilia of the *Chlamydomonas ida3* mutant (6, 20). IDA3 moves by IFT and the levels of IDA I1/f are reduced in a *Chlamydomonas* mutant lacking the IFT-B subunit IFT56 suggesting that this protein is involved in IDA3 mediated ciliary trafficking of IDA I1/f (20, 39). The novel direct interaction between CrIDA3 and CrODA16 uncovered in our AlphaPulldown screen suggests that these two IFT adaptor proteins may collaborate to regulate the trafficking of axonemal dynein arms into cilia. CrIDA3 is an 875-residue protein that is predicted to be mostly disordered, but the N-terminal 1 to 365 residues adopt an α-helical structure interspaced by several loop regions including one long disordered loop between residues 160 to 249. The association with CrODA16 is predicted to occur *via* one loop region (residues 105–116) and two neighboring α-helices (residues 329–362) of CrIDA3 (Fig. 3*A*). Interacting regions are associated with high pLDDT and low PAE values indicative of a high confidence model for the CrODA16-IDA3 complex (Fig. S3*C*). In the structural model of the CrODA16-IDA3 complex, the two helices of IDA3 bind across the β-propeller of CrODA16 and engage in both hydrophobic and hydrophilic contacts with loop regions of the C-terminal face of the CrODA16 β-propeller.

To investigate this interaction experimentally, we recombinantly expressed and purified a CrIDA3 construct containing residues 1 to 166 and 241 to 366 (referred to as CrIDA3 in text and figures), which constitutes the predicted ODA16 binding regions, and assessed binding to CrODA16 in pull-downs. The results show that GST-tagged CrIDA3 pulls down full-length CrODA16 (CrODA16fl) and the CrODA16 C-terminal β-propeller (CrODA16C) but not the small N-terminal domain (CrODA16N) (Fig. 3*B*). We addressed the direct interaction between CrODA16 and CrIDA3 quantitatively using ITC, which revealed a stoichiometric complex with a Kd of 1.77 ± 0.28 μM (Fig. 3*C*). Interestingly, CrODA16C containing only the β-propeller binds CrIDA3 with significantly higher affinity (Fig. S3*B*, Kd = 0.288 ± 0.021 μM, >5 SD below the Kd of CrODA16fl). This result suggests that the presence of the N-terminal ODA16 domain lowers the affinity of the C-terminal β-propeller for IDA3. Complex formation was further confirmed by SEC of CrIDA3 and CrODA16C, which co-eluted as a stable complex (Fig. 3*E*). To experimentally investigate the importance of the two ODA16-associating helices of CrIDA3, an IDA3 construct was purified where the CrODA16-interacting helices were removed (named CrIDA3ΔC in Fig. 3*D*). The result in Figure 3*D* shows that the two α-helices of IDA3 are necessary for the interaction with ODA16. In summary, we discovered that IDA3, an IFT adaptor protein for ciliary trafficking of IDA I1/f, is a direct interactor of the ODA adaptor protein ODA16 in *Chlamydomonas*.

### IDA3 associates with the **β**-propeller of ODA16 to displace IFT46

**Figure 4:**
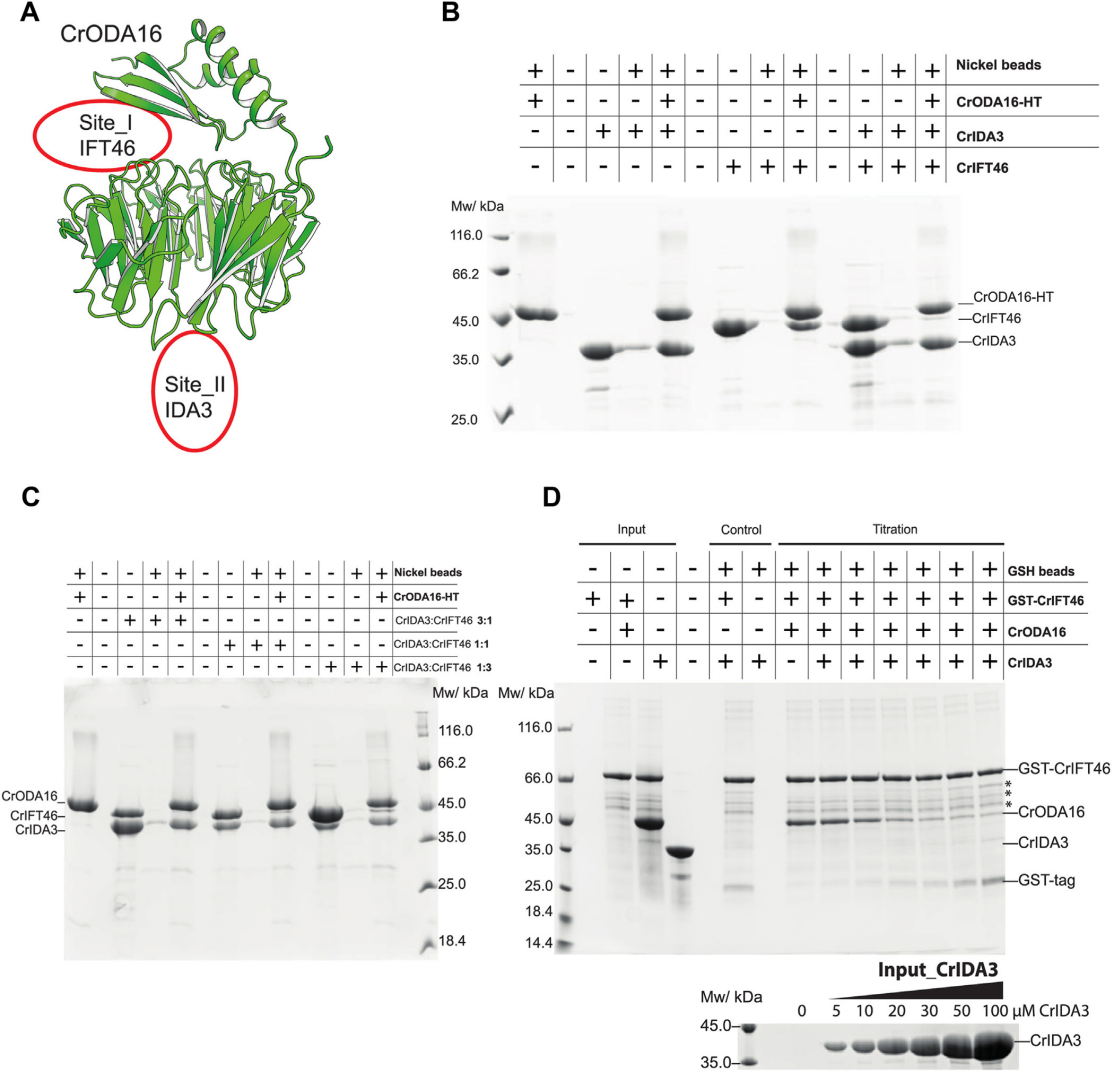
**CrIDA3 and CrIFT46 binding to CrODA16 is mutually exclusive, with CrIDA3 displacing CrIFT46.***A*, AlphaFold 3 structural model of CrODA16 highlighting the distinct binding sites for CrIFT46 (Site I) and CrIDA3 (Site II). *B*, nickel pull-down assay with immobilized CrODA16-HT. CrIFT46 and CrIDA3 bind strongly to CrODA16 individually but not simultaneously. CrIDA3 presence prevents CrIFT46 binding to CrODA16. *C*, Nickel pull-down assay demonstrating weak CrIFT46 binding to CrODA16 in the presence of CrIDA3, even with a 3:1 excess of CrIFT46 to CrIDA3. *D*, titration assay. *Upper panel*: SDS-PAGE of elutions showing CrODA16 dissociation from immobilized GST-CrIFT46 with increasing CrIDA3 concentration (0–100 μM, up to 10-fold excess). *Asterisks* indicate impurities. *Lower panel*: CrIDA3 input amounts used in the titration.

Having established the direct interaction between IDA3 and ODA16, we next explored the functional implications of this association, particularly its impact on the ODA16-IFT46 complex. CrIDA3 and CrIFT46 associate at opposite sides of the CrODA16 β-propeller without any apparent steric clashes (Fig. 4*A*). However, the fact that the ODA16 N-terminal domain lowers the affinity of ODA16 for IDA3 may suggest allosteric communication between the two binding sites highlighted in Figure 4*A*. To further explore this notion, His-tagged CrODA16 was mixed with both CrIFT46 and CrIDA3 revealing that only IDA3 was efficiently pulled down (Fig. 4*B*). This was observed even when CrIFT46 was present in three times molar excess to CrIDA3 (Fig. 4*C*), which suggests that the binding of IDA3 to ODA16 can lower the affinity for IFT46. To assess if CrIDA3 can disrupt a pre-formed CrIFT46-ODA16 complex, a titration assay was performed where purified GST-CrIFT46-ODA16 complex was immobilized on GSH resin and increasing amounts of CrIDA3 (0–100 μM) was added. As shown in Figure 4*D*, CrODA16 was gradually displaced from immobilized GST-CrIFT46 with increasing concentration of CrIDA3. To test if the reverse is also the case, we immobilized GST-CrIDA3-ODA16 on GSH resin and titrated the complex with 0 to 200 μM CrIFT46 (Fig. S4*A*). CrIFT46 was only able to disrupt the CrODA16-IDA3 complex at the very highest CrIFT46 concentrations, and even at 200 μM CrIFT46, the CrODA16-IDA3 complex was only partially disrupted (Fig. S4*A*). Furthermore, SEC analysis of a mixture containing the CrIFT46/56 complex, CrIDA3, and CrODA16 revealed two distinct peaks: one peak where CrODA16 co-eluted with CrIFT46/56 and another peak where CrODA16 co-eluted with CrIDA3 (Fig. S4*B*). Notably, no tetrameric complex was formed, supporting the mutually exclusive binding of CrIDA3 and CrIFT46 on CrODA16. Surprisingly, we were unable to detect a direct interaction between CrIDA3 and CrIFT56/46 even in the absence of CrODA16 (data not shown). We conclude that although IFT46 and IDA3 attach to ODA16 on opposite sides of its β-propeller structure, the binding is mutually exclusive, and IDA3 can displace ODA16 from IFT46. IDA3 is thus unlikely to travel on IFT trains through the interaction with ODA16, but IDA3 may function in the release of ODA16 from the IFT machinery inside cilia.

### Arl3 binds directly to the ODA16 **β**-propeller in a nucleotide-independent manner

AlphaPulldown predicted that both Hs and Cr Arl3, but no other small GTPase present in the 1000 flagellar prey proteins, bind directly to ODA16 (Fig. 2*A*). Arl3 is known to function in the release of farnesylated ciliary proteins (INPP5E, Rheb, and GRK1) from the carrier protein PDE6δ and myristoylated proteins (NPHP3, Cystin-1, ODR-3, and GPA-13) from the carrier protein UNC119 (22, 40, 41). It is also known that Arl13, another ciliary GTPase, is a GEF required for the activation of Arl3 through GDP→GTP nucleotide exchange (25). *Chlamydomonas* Arl3 plays an important role in ciliary protein trafficking. Similar to Arl13, loss of Arl3 causes accumulation of certain membrane-associated proteins like phospholipase D (PLD) in cilia, while reducing levels of others such as FAP12 (42). Arl3 functions together with Arl13b to regulate BBSome-dependent cargo export from cilia. Specifically, Arl3 in its GTP-bound form recruits the PLD-laden BBSome to facilitate its movement across the transition zone for ciliary retrieval (43). This Arl3-Arl13b pathway is critical for maintaining proper levels of signaling proteins in cilia (44). Additionally, active GTP-bound Arl3 functioning in *T. brucei* was reported to displace ODA16 from IFT trains, as ODA16 accumulates in cilia with dominant negative Arl3(T30N) (27). However, there is currently no biochemical explanation for how Arl3 disrupts the interaction between ODA16 and IFT trains.

**Figure 5:**
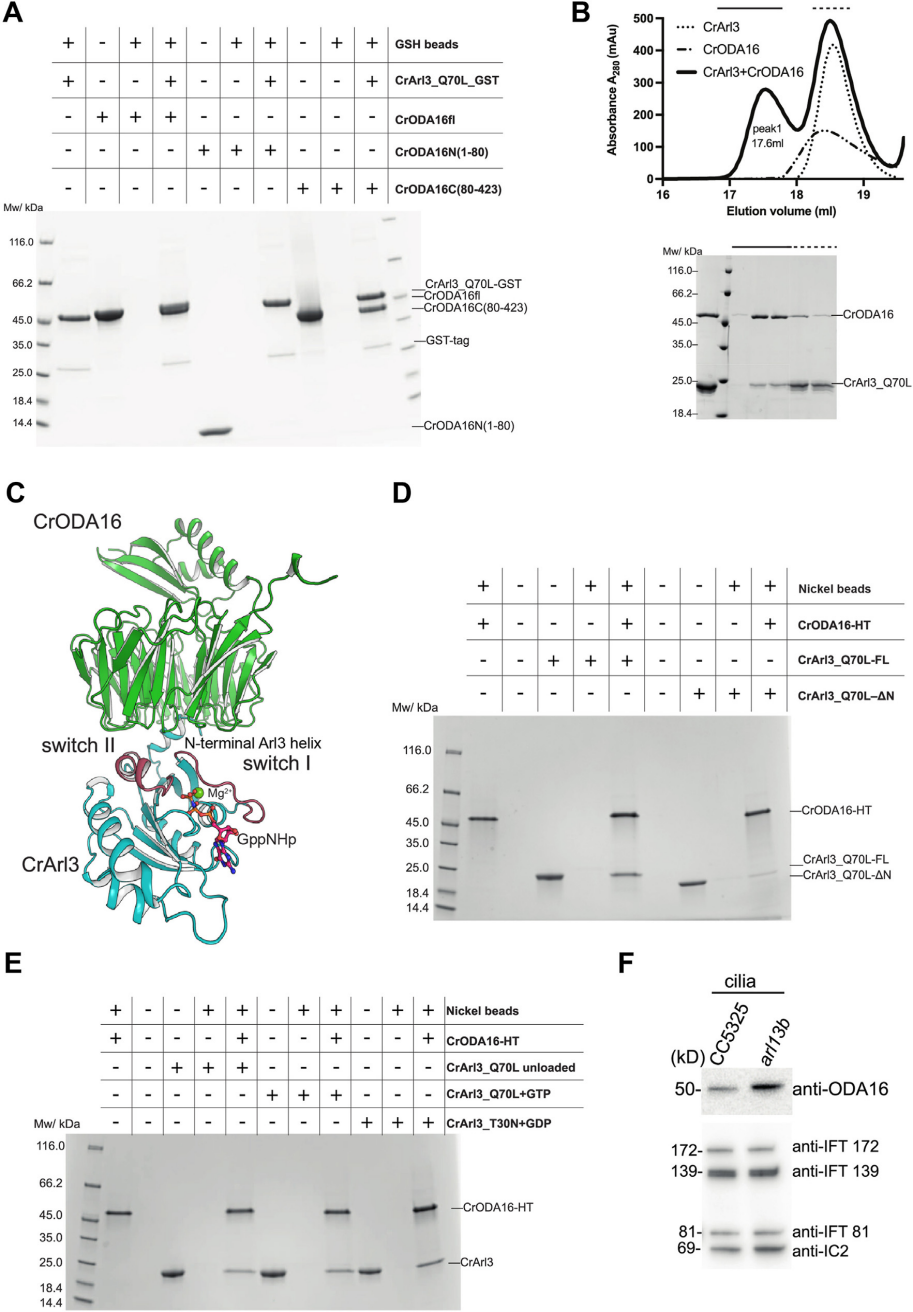
**CrArl3 N-terminal helix directly interacts with CrODA16 β-propeller.***A*, GSH pull-down assay with immobilized GST-CrArl3 demonstrating direct binding between CrArl3 and CrODA16. Full-length CrODA16 (CrODA16fl) and its β-propeller domain (CrODA16C(80–423)) bind to CrArl3, while the N-terminal fragment (CrODA16N(1–80)) does not. *B*, size exclusion chromatography (SEC) profile (*upper panel*) of CrODA16 incubated with CrArl3. The corresponding SDS-PAGE analysis of SEC fractions (*lower panel*) confirms the formation of a stable CrODA16-Arl3 complex in peak 1. *C*, alphaFold-predicted model of the CrArl3 (*cyan*) and CrODA16 (*green*) complex. The interaction primarily involves the N-terminal α-helix of CrArl3 and the CrODA16 β-propeller. Switch regions of CrArl3 are shown in *red-brown*. The magnesium ion (*yellow*) and GppNHp are positioned based on alignment with the experimental CrArl3 structure (PDB: 5DE3). *D*, nickel pull-down assay with immobilized CrODA16-HT. The CrArl3 truncation lacking the N-terminal α-helix (CrArl3_Q70L-ΔN) shows significantly reduced binding to CrODA16 compared to full-length CrArl3 (CrArl3_Q70L-FL). *E*, nickel pull-down assay with immobilized CrODA16-HT demonstrating that CrODA16 binds CrArl3_Q70L regardless of GTP presence and CrArl3_T30N with GDP, indicating nucleotide-independent binding between CrODA16 and CrArl3. *F*, Western blot analysis using anti-CrODA16 antibodies shows increased accumulation of CrODA16 in *arl13b Chlamydomonas* cilia compared to wild-type (CC5325) cilia. Anti-IFT172, anti-IFT139, anti-IFT81, and anti-IC2 antibodies were used as loading controls.

To investigate experimentally if there is a direct interaction between Arl3 and ODA16, and to study the regulatory mechanism, CrArl3 was recombinantly produced and purified as GTP-locked Q70L and GDP-locked T30N variants. Recombinant CrArl3_Q70L was highly pure (Fig. S5*A*) and did not carry over nucleotides during the purification (Fig. S5*B*). Similar to CrIDA3, the purified CrArl3_Q70L variant also binds directly to both CrODA16fl and CrODA16 β-propeller but not to CrODA16N in pull-down experiments (Fig. 5*A*). CrArl3 and CrODA16 were also verified to form a stable complex in SEC (Fig. 5*B*). The CrODA16-Arl3 interaction was predicted with very high confidence using AlphaFold (Fig. S5*C*) and is mediated mainly by the N-terminal α-helix of Arl3, which inserts into the central channel of the ODA16 β-propeller (Fig. 5*C*). Additional contacts occur between switch regions I and II of Arl3 (colored red-brown in Fig. 5*C*) and residues located in loops of the ODA16 β-propeller (Fig. 5*C*). We note that the binding sites for Arl3 and IDA3 on ODA16 overlap and are predicted to be mutually exclusive (Fig. S5*D*). Consistent with the AlphaFold model, the removal of the N-terminal helix of CrArl3 (CrArl3_Q70L-ΔN in Fig. 5*D*) resulted in a strong reduction in binding to CrODA16 (Fig. 5*D*).

In the article from Huang and colleagues, it is reported that only the active GTP-loaded but not GDP-loaded TbArl3 can displace TbODA16 from the IFT train *in vivo* (27). The predicted structural model of the CrArl3-ODA16 complex shows that the switch regions I and II of CrArl3 adopt the active GTP-bound conformation (Fig. 5*C*, GppNHp∗Mg^2+^ modeled from the crystal structure of Arl3; PDB code 5DE3). The structural prediction suggests that mainly the N-terminal helix and to a lesser degree switch regions I and II of CrArl3 are directly involved in CrODA16 binding. To test if the binding between recombinant CrArl3 and CrODA16 is dependent on the nucleotide state of CrArl3, both the inactive CrArl3_T30N and the active CrArl3_Q70L variants were used for binding assays in the presence of GDP or GTP, respectively. According to the pull-down results shown in Figure 5*E*, both the inactive CrArl3_T30N_GDP, the active CrArl3_Q70L_GTP, and the nucleotide empty CrArl3_Q70L_unloaded bind CrODA16 in pull-down experiments. The direct interaction between CrArl3 and CrODA16 *in vitro* is thus not influenced by the nucleotide state of CrArl3.

What may the reason be for the apparent discrepancy in nucleotide dependence for Arl3 binding to ODA16 besides species-specific function in Cr *versus* Tb? The N-terminal α-helix of Arl3 involved in the binding of ODA16 is an amphipathic helix important for ciliary localization and membrane association (22, 23, 42, 45). The deletion of the Arl3 N-terminal α-helix results in a complete loss of ciliary localization and membrane association of Arl3 (23, 42). In *Chlamydomonas*, the inactive GDP-locked Arl3 remains associated with the ciliary membrane *via* this N-terminal α-helix, while the active Arl3 is soluble in the flagellar matrix (42). It is thus possible that inactive Arl3 cannot displace ODA16 from IFT trains *in vivo* simply because the N-terminal α-helix of Arl3 is engaged with the ciliary membrane and thus inaccessible for ODA16 interaction. A similar mechanism could occur in Tb preventing GDP-loaded TbArl3 from interacting with TbODA16.

In agreement with this notion, when TbArl13b, which is a GEF for TbArl3, is knocked out, TbODA16 accumulates in cilia (27). To examine if the same mechanisms are at play in *Chlamydomonas*, we grew WT CC5325 and *arl13b* null-mutant *Chlamydomonas* strains and examined the ciliary amounts of ODA16. The result shows a clear accumulation of ODA16 in *arl13b* null-mutant cilia compared to control *Chlamydomonas* cells despite similar levels of IFT-A and IFT-B proteins (Fig. 5*F*). This result supports the notion that CrArl3 plays a role in releasing CrODA16 from IFT trains in *Chlamydomonas*, similar to what was reported for *T. brucei*. Having characterized the direct interaction between Arl3 and ODA16, we next sought to understand how this interaction might influence the association of ODA16 with the IFT machinery.

### Arl3 displaces IFT46 from ODA16

**Figure 6:**
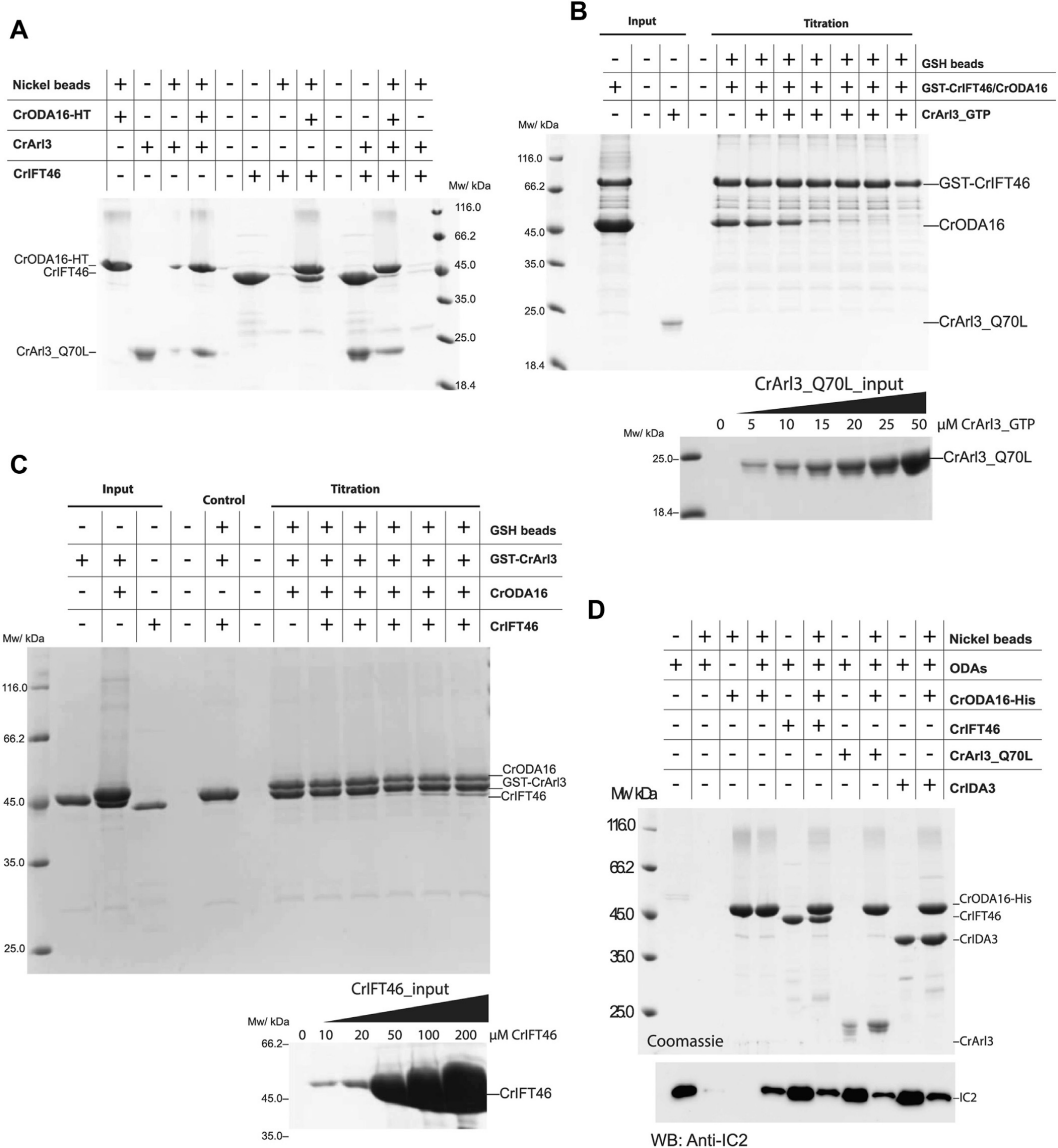
**CrArl3 binding to CrODA16 disrupts CrIFT46 interaction but does not fully release ODAs.***A*, pull-down assay with immobilized CrODA16-HT (his-tagged) shows mutually exclusive binding of CrIFT46 and CrArl3 to CrODA16. CrArl3 presence prevents CrIFT46 binding to CrODA16. *B*, titration assay using GSH-immobilized GST-CrIFT46-ODA16 complex. Increasing CrArl3 concentrations (0–50 μM) causes CrODA16 to dissociate from GST-CrIFT46. *Upper panel* shows pull-down results; *lower panel* shows CrArl3 input. *C*, titration assay with GSH-immobilized GST-CrArl3-ODA16 complex. CrODA16 remains bound to GST-CrArl3 even with high CrIFT46 concentrations (up to 200 μM). *Upper panel* shows pull-down results; *lower panel* shows CrIFT46 input. *D*, pull-down from *Chlamydomonas* flagellar axoneme fractions using His-tagged CrODA16 alone or in complexes. ODAs (detected by anti-IC2 antibody) remain associated with CrODA16 in all cases, with slight dissociation observed when CrArl3 is present. *Upper panel*: Coomassie-stained SDS-PAGE; *lower panel*: Western blot for IC2.

The AlphaFold models predict that both CrArl3 and CrIDA3 use α-helices to bind the electro-negative surface of the CrODA16 β-propeller (Fig. S5*D*) without overlap with the CrIFT46-binding site. However, when both CrArl3 and CrIFT46 are present in a pull-down experiment, CrODA16 binds exclusively to CrArl3 (Fig. 6*A*). To determine if CrArl3 can disrupt the CrIFT46-ODA16 interaction, a titration assay was performed with increasing amounts of CrArl3 (0–50 μM). As shown in Figure 6*B*, CrODA16 lost its binding to immobilized GST-CrIFT46 when increasing amounts of CrArl3 were added. Conversely, when GST-CrArl3-ODA16 was immobilized on GSH resin, CrODA16 remained associated with immobilized GST-CrArl3, even in the presence of a 20 times molar excess (200 μM) of CrIFT46 (Fig. 6*C*). These findings demonstrate that CrArl3 binding to CrODA16 can disrupt the CrIFT46-ODA16 interaction, but CrIFT46 cannot displace CrArl3 from CrODA16. The ability of CrArl3 to displace CrIFT46 from CrODA16 is consistent with the observations reported in *T. brucei* (27) and suggests that the release of ODA16 from IFT trains is likely a direct consequence of Arl3 binding to ODA16 and displacing it from IFT46.

Structural modeling suggests that CrIDA3 and CrArl3 bind to overlapping sites on CrODA16, indicating their binding is likely mutually exclusive (Fig. S5*D*). Pull-down experiments demonstrated that the binding of CrIDA3 and CrArl3 to CrODA16 is competitive and depends on their relative concentrations in the system (Fig. S6, *A* and *B*). Since the binding of CrArl3 and to a lesser degree CrIDA3 to CrODA16 can disrupt the CrODA16-IFT46 interaction and likely displace CrODA16 from IFT trains, we wanted to investigate if ODAs are also released from CrODA16 in this process. ODAs were isolated from *Chlamydomonas* flagella and pull-down assays were performed to probe for the association of ODAs with CrODA16 in the absence or presence of CrIDA3 or CrArl3. His-tagged CrODA16 was immobilized on Ni^2+^-NTA resin and incubated with axonemal extracted ODAs with or without a 4-fold molar excess of CrIFT46, CrArl3, or CrIDA3. The presence of co-immunoprecipitated ODAs in each reaction was probed using a mouse monoclonal antibody against the IC2 subunit of ODAs (46). As shown in Figure 6*D*, ODAs associate with CrODA16 in the absence or presence of CrIFT46, consistent with previous reports that ODA16 acts as an adaptor for loading ODAs onto IFT trains (14, 15). Interestingly, the presence of CrArl3 slightly reduces the amount of ODAs bound to CrODA16 suggesting that CrArl3 initiates the process of ODA release from ODA16. However, the incomplete dissociation indicates that additional factors are likely required for full ODA release and subsequent incorporation into the axoneme.

## Discussion

Outer dynein arms require synthesis, assembly, and transport before docking onto their site of function in the axoneme, and these steps rely on different adaptor proteins and complexes whose exact interactions and regulatory mechanisms are not fully explored. In this study, we combined structural modeling with biochemistry of human and *Chlamydomonas* proteins to detect several novel interactions and regulatory mechanisms for the ciliary trafficking of ODAs and the release of the ODA16 cargo adaptor from the IFT machinery. Based on these findings, we provide insights into how ODA trafficking may be orchestrated by ODA16, and IFT46 and regulated by Arl3 and IDA3.

Our study identifies an NxxxD(E)E motif in the N-terminus of IFT46 that is critical for its interaction with ODA16 (Fig. 1, *A*–*D*). This motif appears to be conserved across several species with motile cilia that rely on intraflagellar transport (IFT), suggesting a common mechanism for linking ODAs to the IFT machinery *via* ODA16 (Fig. S1*B*). However, this motif is notably absent in mammalian IFT46 homologs, indicating potential species-specific differences in ODA transport mechanisms (19).

### *Chlamydomonas* IDA3 releases ODA16 from IFT46

A key finding of our study is the discovery that IDA3, previously known for its role in inner dynein arm transport (20), can directly interact with ODA16 to disrupt the interaction of ODA16 with IFT46 (Figs. 3, *A*–*C*, 4, *A*–*D*). This interaction occurs at a distinct site on the ODA16 β-propeller, separate from the IFT46 binding site. Despite this spatial separation, IDA3 binding appears to allosterically reduce the affinity of ODA16 for IFT46, suggesting a potential mechanism for regulating ODA release from IFT trains. This IDA3-mediated displacement of ODA16 from IFT46 raises intriguing questions about the coordination of outer and inner dynein arm transport. Our results suggest a possible crosstalk between the IFT of ODA and IDA. Given that IDA3 is an essential and selective IFT adapter for IDA I1/f, and its ciliary distribution is regulated by ciliary length (20), this crosstalk likely occurs at specific stages, such as during the initiation of ciliogenesis.

It is possible that IDA3 plays a dual role in both transporting IDA and regulating ODA delivery within the cilium. IDA3 is known to traffic on anterograde IFT trains in a ciliary length-dependent manner (20). Given that IDA3's association with ODA16 dissociated ODA16 from IFT46, it appears unlikely that IDA3 travels on anterograde IFT trains as a cargo of ODA16. IDA3 may travel on anterograde IFT trains independent of ODA16 and, once IDA3 is released, help release ODA16 and associated ODA cargoes from IFT trains. Although our findings contribute to a more nuanced understanding of dynein arm trafficking and highlight the complex interplay between adaptor proteins in this process, further investigation is needed to understand the precise timing and localization of these interactions *in vivo*.

### Arl3 binds directly to ODA16 to release it from the IFT machinery

Our investigation reveals a direct interaction between the N-terminal amphipathic α-helix of CrArl3 and CrODA16 (Fig. 5, *A*–*D*). The nucleotide state of Arl3 does not impact the direct interaction with ODA16 *in vitro* (Fig. 5*E*) but might be important for Arl3 membrane association, which likely indirectly regulates the access of Arl3 to ODA16 in cilia. Notably, recent literature presents conflicting views on Arl3 nucleotide-dependent membrane association. While one study suggested that Arl3-GTP but not Arl3-GDP binds membranes (45), another study reported Arl3-GDP can also be associated with membranes, particularly when using *E. coli* total lipid extracts (42). These divergent findings highlight the potential complexity of Arl3 membrane interactions, which may be critically dependent on specific lipid compositions that mimic the native ciliary membrane environment. Importantly, Arl3 binds to the C-terminal face of the ODA16 β-propeller (Fig. 5*C*), a site distinct from the IFT46 binding cleft between the N- and C-terminal domains of ODA16 (Fig. 1*A*) (17). Intriguingly, we find that titrating the ODA16-IFT46 complex with Arl3 dissociates ODA16 from IFT46 suggesting that Arl3 can release ODA16 from IFT trains (Fig. 6*B*). These findings are consistent with reports in *T. brucei* showing that Arl3 facilitates ODA16 unloading from IFT trains in cilia (27). We also observed a clear accumulation of ODA16 in *arl13b* null-mutant *Chlamydomonas* cilia (Fig. 5*F*), supporting a conserved function of Arl3 in regulating ODA transport across ciliated organisms. Interestingly, it thus appears that in addition to its well-described role in the release of lipidated ciliary cargoes from carrier proteins, Arl3 has also evolved to release ODA16 from the IFT machinery.

How may Arl3 association with ODA16 far away from the IFT46 binding site regulate dissociation of ODA16 from IFT trains? *In vitro* pull-down and titration, assays demonstrated that CrArl3 binding to CrODA16 disrupts the CrIFT46 association (Fig. 6, *A* and *B*). This finding supports the hypothesis that Arl3 directly mediates the release of ODA16 from IFT trains in cilia. Intriguingly, the different binding sites of CrArl3 and CrIFT46 on CrODA16 suggest an allosteric mechanism of release rather than a direct steric hindrance. We propose that CrArl3 binding to the C-terminal face of the β-propeller induces conformational changes in CrODA16, which in turn disrupt its interaction with CrIFT46 unloading ODAs from IFT trains. However, it is important to note that while Arl3 effectively displaces ODA16 from IFT46, it does not appear to fully release ODAs from ODA16 (Fig. 6*D*). This observation suggests that additional factors or steps may be involved in the final release and docking of ODAs onto the axoneme. Future experimental structural studies will be crucial to elucidate the precise mechanism of this allosteric regulation and to understand how it integrates with other aspects of ODA trafficking and assembly.

### Regulation of ODA16-mediated transport by IDA3 and Arl3

**Figure 7:**
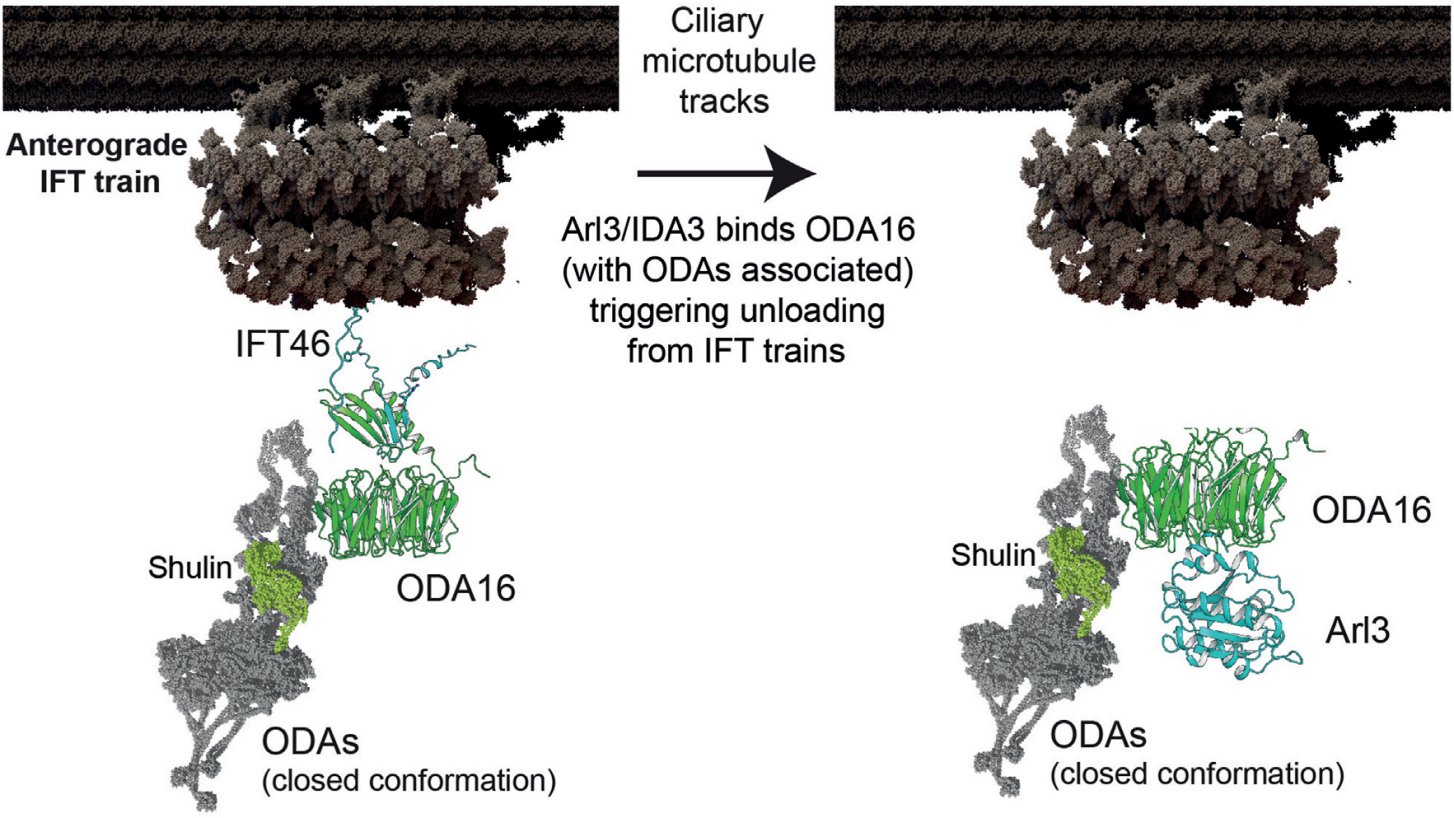
**Schematic model for Arl3-mediated release of ODA16 from IFT trains.***Left panel*: An anterograde IFT train (adapted from (53)) moving along ciliary microtubules carries ODA16 (*green*) bound to IFT46 (*cyan*). ODA16 associates with Shulin (*lime*) bound ODAs (*grey*) in their closed conformation (adapted from (9)). The relative positions of ODA16 on the IFT train and its association with ODAs are not precisely known. *Right panel*: Binding of Arl3 (*cyan*) to ODA16 triggers release from IFT46 while maintaining ODA association. Note that protein structures are not shown to scale; ODA16 and IFT46 are enlarged for clarity. The model is a mixture of *Chlamydomonas* and *Tetrahymena* structures and is not meant to represent a precise structural model.

Our study reveals that both IDA3 and Arl3 can regulate ODA16-mediated transport by triggering release from IFT46. Intriguingly, while both proteins bind to sites on the ODA16 β-propeller that are distinct from the IFT46 binding interface, they can effectively displace ODA16 from IFT46, suggesting possible allosteric regulation. The existence of two proteins that can trigger this release through binding to non-overlapping sites with IFT46 may allow cells to precisely control ODA delivery in different contexts - Arl3 providing a conserved regulatory system across ciliated organisms, while IDA3 may enable *Chlamydomonas* to coordinate outer and inner dynein arm assembly. Importantly, our biochemical data reveal that while both IDA3 and Arl3 can effectively trigger the release of ODA16 from IFT trains, neither fully releases ODAs from ODA16 (Fig. 6*D*). This suggests that the release mechanism specifically affects the ODA16-IFT46 interface, while ODA release and axonemal integration likely require additional factors and regulatory steps. The persistence of ODA binding after IDA3/Arl3-induced release from IFT trains may serve as a crucial checkpoint, ensuring ODAs remain associated with their adaptor until reaching appropriate sites for axonemal assembly (Fig. 7). This regulated release of ODA16 from IFT trains represents a key step in ODA delivery and works in concert with other regulatory mechanisms - for example, the assembly factor Shulin maintains ODAs in a closed conformation during transport to prevent premature interactions with microtubules (9). The conservation of this regulatory mechanism, particularly through Arl3, across diverse ciliated organisms highlights its fundamental importance in ciliary transport. Our structural modeling and biochemical analyses provide key insights into how cells can achieve precise control over the offloading of essential axonemal components during ciliary assembly through the regulated release of adaptor-transport machinery interactions.

## Experimental procedures

### AlphaPulldown and structural modeling

AlphaPulldown, an *in silico* screening method for protein-protein interactions (PPI) based on the AlphaFold-Multimer algorithm, was used to predict potential interactors of CrODA16 and HsODA16. The AlphaPulldown software package (31) was employed to streamline the PPI screens and high-throughput modeling of protein complexes. The top 1000 entries from curated databases of *Chlamydomonas* and human flagellar proteins (33) were used as prey proteins. Interaction predictions were ranked according to the combined predicted template modeling (pTM) and interface predicted template modeling (ipTM) scores provided by AlphaPulldown. See Table S1 for a sorted list of potential ODA16 interactors. Hits with a combined pTM/ipTM score above 0.6 were further analyzed using AlphaFold 3 (30) to generate refined structural models of the protein complexes. The PAE plots, ipTM and pLDDT scores were used to assess the confidence of the structural predictions. PAE plots were generated using the PAE viewer tool (47). All structural visualizations were performed using PyMOL (Schrödinger, LLC, https://pymol.org) or UCSF Chimera (48). Electrostatic surface potentials were calculated using the APBS plugin in PyMOL.

### Cloning, protein expression, and purification

CrIDA3 and CrArl3 genes were codon-optimized and synthesized commercially (Genscript). CrArl3 was cloned into pEC_A with C-terminal TEV-His8 or TEV-GST-His8 tags for bacterial expression. Inactive (T30N) and active (Q70L) variants were generated, along with a truncated Arl3_Q70L_ΔN lacking the N-terminal 17 amino acids. CrIDA3 construct, containing structured fragments containing residues (1–166) and (241–366), was cloned into pEC_A with N-terminal His6-TEV or His6-GST-TEV tags for bacterial expression. A truncation (CrIDA3ΔC) lacking residues (333–366) where the CrODA16-interacting helices are located was also generated. HsIFT81/74 N-terminal complex used in Figure 2*B* was expressed and purified as previously detailed (49). HsODA16 WT and N143D mutants were expressed in insect cells as previously reported (19). CrODA16fl, CrODA16N(1-80), and CrODA16C(80-423) were expressed in insect cells following established protocols (17). Protein purification procedures followed those previously described for each respective construct (17, 19), with minor modifications as needed for specific proteins.

### Pull-down assays

Pull-down assays were performed to investigate PPIs using GST-tagged, His-tagged, or GFP-tagged bait proteins. Pull-down assays were performed using binding and wash buffers containing 200 mM NaCl to maintain consistency with previous structural and biochemical studies of ODA16. While this concentration is higher than physiological conditions for *Chlamydomonas* (25–50 mM range (50)), the robust interactions we observe under these more stringent conditions support their physiological relevance. Bait proteins (10 μM) were immobilized on appropriate affinity resins (GSH, nickel, or GFP-Binder beads) by incubation for 1 h at 4 °C. The resin was then washed three times with binding buffer (10 mM HEPES pH 7.5, 200 mM NaCl, 2 mM MgCl_2_ and 1 mM DTT). Prey proteins (20–30 μM) were added to the immobilized bait and incubated for 1 h at 4 °C. After washing three times with binding buffer, bound proteins were eluted using tag-specific methods: 33 mM reduced glutathione for GST-tagged proteins, 500 mM imidazole for His-tagged proteins, or 0.1 M citric acid (neutralized with Tris-base) for GFP-tagged proteins. For GFP-tagged proteins, cell lysates from HEK293s cells expressing the bait protein were used instead of purified protein. Eluted samples and inputs were analyzed by SDS-PAGE. Control experiments using empty resin were performed to detect non-specific interactions. Protein concentrations and volumes were adjusted as needed for each experiment.

### Competitive binding assays

To investigate the ability of CrArl3 and CrIDA3 to disrupt the ODA16/IFT46 interaction, we performed competitive binding assays. GST-tagged CrIFT46 (10 μM) was mixed with CrODA16 (20 μM) in binding buffer (total volume 100 μl) and incubated with 25 μl of GSH resin for 1 h at 4 °C. After washing the resin three times with 150 μl of binding buffer, untagged CrArl3 or CrIDA3 at various concentrations (in 300 μl binding buffer) was added to the immobilized GST-CrIFT46/CrODA16 complex. Following a 1-h incubation at 4 °C, the resin was washed three times with binding buffer, and bound proteins were eluted with 30 μl of elution buffer (binding buffer containing 33 mM reduced glutathione). Eluted samples were analyzed by SDS-PAGE.

For the reciprocal experiment testing IFT46's ability to disrupt CrArl3/CrODA16 or CrIDA3/CrODA16 interactions, GST-tagged CrArl3 or CrIDA3 (10 μM) was first complexed with CrODA16 (20 μM) and immobilized on GSH resin. After washing, the immobilized complexes were incubated with 300 μl of untagged CrIFT46 at various concentrations. Subsequent washing, elution, and analysis steps were performed as described above. All incubations were carried out on a rotating wheel in a cold room. Centrifugation steps for resin collection were performed at 800*g* for 3 min at 4 °C. Input samples were collected before each assay for SDS-PAGE analysis.

### Isothermal titration calorimetry

PPIs were quantitatively analyzed using ITC, following the method described by Taschner *et al.* (17) with minor modifications. All experiments were conducted at 25°C using a MicroCal iTC200 (MicroCal, Malvern Panalytical). The ITC buffer contains 10 mM HEPES (pH 7.5), 200 mM NaCl, and 0.5 mM TCEP. For CrODA16-IFT46 peptide and CrODA16-CrIDA3 interactions, the sample cell contained 200 μl of CrODA16 at a concentration of 20 μM. The injection syringe was loaded with either IFT46 peptide or CrIDA3 at a concentration of 200 μM. Titrations were performed by sequential injections of 2 μl of the syringe content into the sample cell with a stirring speed of 1000 rpm. The obtained ITC data were analyzed using the Origin 7 software provided by MicroCal (Malvern Panalytical) to determine thermodynamic parameters, including the dissociation constant (Kd), enthalpy change (ΔH), and stoichiometry of the interaction.

### Isolation of outer dynein arms and CrODA16 pull-down assays

*Chlamydomonas* flagella were isolated from 8 L of *Chlamydomonas* culture (CC-1690) using dibucaine (51). Purified flagella were resuspended in HMDEK buffer (30 mM HEPES, 5 mM MgSO_4_, 1 mM DTT, 0.5 mM EGTA, and 25 mM KCl) and lysed by three freeze-thaw cycles. The axonemal fraction was separated by centrifugation (10,000 rpm, 10 min). ODAs were extracted from the axonemal pellet using HMDE buffer (30 mM HEPES, 5 mM MgSO_4_, 1 mM DTT, and 0.5 mM EGTA) containing 0.6 M KCl. After 30 min incubation on ice and centrifugation (13,000 rpm, 20 min), the supernatant containing ODAs was dialyzed overnight in HMDEK buffer with 50 mM KCl.

For pull-down assays, His-tagged CrODA16 (10 μM) was immobilized on nickel resin in binding buffer (20 mM HEPES, pH 7.5, 200 mM NaCl, 1 mM MgCl_2,_ and 1 mM DTT). ODA extract (200 μl) was applied to CrODA16-immobilized resin or control resin. To assess the influence of CrIFT46, CrIDA3, or CrArl3 on ODA binding, these proteins (40 μM) were pre-incubated with ODA extracts before addition to immobilized CrODA16. After 4 h incubation at 4 °C, the resin was washed three times with binding buffer, and bound proteins were eluted with 500 mM imidazole. Eluted samples were analyzed by SDS-PAGE and Western blotting. Membranes were probed with anti-IC2 primary antibody (1:500 (46)) and HRP-conjugated secondary antibody (1:1000; Dako). Signals were detected using ECL Prime reagent (Amersham) and imaged with a ChemiDoc system (BioRad).

### Nucleotide loading of CrArl3

Purified CrArl3_Q70L or CrArl3_T30N (20 mg) was buffer exchanged into SEC buffer (10 mM HEPES pH 7.5, 150 mM NaCl, and 1 mM DTT) and concentrated to 1 ml. A 10-fold molar excess of GDP was added, and the mixture was incubated at 20 °C for 30 min. EDTA was then added to a final concentration of 10 mM, and the sample was incubated at 20 °C for 3 h. Nucleotide-free CrArl3 was isolated by SEC using a Superdex 75 increase 10/300 Gl column equilibrated in SEC buffer. For binding assays, nucleotide-free CrArl3 variants were reloaded with specific nucleotides. CrArl3_T30N was supplemented with 1 mM MgCl_2_ and 1 mM GDP to generate GDP-loaded CrArl3_T30N. CrArl3_Q70L was supplemented with 1 mM MgCl_2_ and either 1 mM GTP or 1 mM GTPγS to generate GTP-loaded CrArl3_Q70L. The nucleotide-free state of Arl3_Q70L was confirmed by HPLC analysis (Fig. S5*B*). This procedure allowed for the preparation of CrArl3 in defined nucleotide-bound states for subsequent binding assays and functional studies.

### Cell culture and western blot analysis of isolated flagella

CC5325 (WT) and *arl13b* mutant (44) strains were grown in modified M medium (https://www.chlamycollection.org/methods/media-recipes/minimal-or-m-medium-and-derivatives-sager-granick/) at ∼23 °C under a 14:10 h light/dark cycle. For cilia isolation, we used 0.5-1L mid-log growth phase cultures aerated with 0.5% CO_2_. Cilia isolation was performed as described by Witman (51) and Lefebvre (52). In brief, cilia shedding was triggered by treatment with dibucaine and vigorous pipetting and cilia were purified and collected by a series of differential centrifugations.

For immunoblotting, cilia samples were mixed 1:1 with 2.5 × SDS sample buffer, incubated at 95 °C for ∼5 min, and proteins were separated by SDS-PAGE using precast gels (Bio-Rad TGX; 4–15%). After electro-transfer to PVDF membranes (Millipore), the membranes were blocked for 1 h in TBS supplemented with 0.05 Tween20 (TBS-T) and containing 3% non-fat milk and 0.5% fish gelatin. Membranes were incubated overnight at 4 °C with gentle agitation in the primary antibodies (anti-IC2, 1:4000 (46); anti-IFT81, 1:500; anti-IFT139, 1:400; anti-IFT172, 1:400 (12) and anti-ODA16, 1:50 (14) in blocking buffer, using replica membranes or sequential staining of the same membrane. Then, the membranes were washed repeatedly in TBS-T, followed by an incubation of 90 min in the secondary antibodies (*i.e.*, anti-mouse and anti-rabbit IgG conjugated to horseradish peroxidase; Invitrogen 31432/AB_228302 and 31460/AB_228341, respectively, diluted in blocking buffer). After repeated washes in TBS-T, the membranes were treated with chemiluminescent substrate (SuperSignal West Pico PLUS or Atto; Thermo Fisher Scientific) and imaged using the Bio-Rad ChemiDoc Touch imaging system and Image Lab 5.2.1 software. The Western blot shown in Figure 5*F* was selected from three biological replicates.

## Data availability

Predicted structural models from AlphaFold 3 are available in ModelArchive (https://modelarchive.org/). The following models have been deposited: CrIFT46/ODA16 (Figs. 1*A* and 2*A*) under model ID ma-byxya; HsIFT46_Cr (22, 23, 24, 25, 26, 27, 28, 29, 30, 31, 32, 33, 34, 35, 36, 37, 38, 39, 40, 41, 42, 43, 44)/ODA16 (Fig. S1*C*) under ma-032z7; CrODA16/Arl3 (Fig. 2*A*) under ma-wzhji; CrODA16/FAP20 (Fig. 2*A*) under ma-23fpw; CrODA16/IDA3 (Figs. 2*A* and 3*A*) under ma-3zu6l; HsODA16/IFT81/IFT74 (Fig. 2*B*) under ma-yblfr; HsODA16/CFA20 (Fig. 2*B*) under ma-pfaor; HsODA16/ODA8 (Fig. 2*B*) under ma-raezb; HsODA16/Arl3 (Fig. 2*A*). These models can be accessed by appending the model ID to the ModelArchive URL, *e.g.*, https://modelarchive.org/doi/10.5452/ma-byxya for the CrIFT46/ODA16 model.

## Supporting information

This article contains supporting information.

## Conflict of interests

The authors declare that they have no conflicts of interest with the contents of this article.
