# Supplementary material for: Integrative in silico and biochemical analyses demonstrate direct Arl3-mediated ODA16 release from the intraflagellar transport machinery
Source: J Biol Chem. 2025 Jan 27;301(3):108237. doi: 10.1016/j.jbc.2025.108237 (PMC11879689; doi:10.1016/j.jbc.2025.108237)
Supplement: Supplemental Material [file mmc1.docx]

Supporting Figures and supporting legends

**Figure S1: Conservation of the IFT46 NXXXD(E)E motif binding pocket in ODA16**

A) AlphaFold model of the CrIFT46-ODA16 complex colored by per-residue pLDDT confidence score (left) and predicted alignment error (PAE) (right). The PAE plot assesses the confidence in the relative position of subunits within the complex. The residues of the corresponding subunits are indexed as indicated. The aligned error in angstroms (Å) is color coded from 0-30Å so that dark green color indicates low PAE (high confidence) and white color indicates high PAE (low confidence). Low PAE values indicate high confidence in the CrIFT46-ODA16 interaction.

B) Multiple sequence alignment of IFT46 homologues from various species. The conserved NXXXD(E)E motif is highlighted with a red line. Species with this motif (Cr-, Tb-, Dr-, Tt-, and Xl-) are predicted to interact with their respective ODA16 homologues, while those lacking it (Hs-, Mm-, and Bt-) may not (data not shown). Species abbreviations: Cr: *Chlamydomonas reinhardtii*; Tb: *Trypanosoma brucei*; Dr: *Danio rerio*; Tt: *Tetrahymena thermophila*; Xl: *Xenopus laevis*; Ce: *Caenorhabditis elegans*; Hs: *Homo sapiens*; Mm: *Mus musculus*; Bt: *Bos taurus.*

C) AlphaFold-predicted model of HsODA16 (green) in complex with the chimeric HsIFT46_Cr construct (cyan) (left). The CrIFT46(22-44) fragment is highlighted in reddish-brown. Close-up view (right) shows polar interactions between HsODA16 and the CrIFT46(22-44) fragment, indicating conservation of the IFT46-binding pocket in HsODA16.

D) AlphaFold model of the HsIFT46_Cr/HsODA16 complex from panel C, colored by per-residue pLDDT confidence score (left) and PAE (right). Low PAE values (darker green) indicate high confidence in the interaction between HsODA16 and the CrIFT46(22-44) fragment.

**Figure S2: Confidence indicators for ODA16 structural models**

Structural models of complexes of ODA16 with *Cr* (A) and Hs (B) interactors colored by per-residue pLDDT confidence score. C) Predicted alignment error (PAE) plots for the structures shown in panels A-B. Darker green indicates higher confidence interactions.

**Figure S3: Detailed characterization of the CrIDA3-CrODA16 interaction**

A) Size exclusion chromatography (SEC) profile of CrIDA3 (top panel) and corresponding SDS-PAGE analysis of SEC fractions (bottom panel). The single peak and consistent band size across fractions indicate that CrIDA3 is monodisperse and stable.

B) Isothermal titration calorimetry (ITC) analysis of CrIDA3 binding to CrODA16C(80-423). The thermogram (top) and binding isotherm (bottom) demonstrate a high-affinity interaction with a Kd of 0.288 ± 0.021 μM and a stoichiometry (N) of 1.06 ± 0.01.

C) AlphaFold 3 prediction of the CrODA16-IDA3 complex structure. Left: 3D model colored by prediction confidence (pLDDT score). Right: PAE plot showing predicted residue-residue interactions between CrODA16 and CrIDA3. Darker green indicates higher confidence interactions.

**Figure S4: CrIFT46 ineffectively competes with CrIDA3 for CrODA16 binding**

A) Titration assay. Upper panel: SDS-PAGE of elutions showing CrODA16 retention on immobilized GST-CrIDA3 with increasing CrIFT46 concentration. CrODA16 begins to dissociate only at very high CrIFT46 concentrations (100-200 μM, 20-fold excess). Lower panel: CrIFT46 input amounts (10-200 μM) used in the titration.

B) Size exclusion chromatography (SEC) analysis of CrIFT46/56 complex incubated with CrIDA3 and CrODA16. Upper panel: SEC profiles of CrIFT46/56+CrIDA3+CrODA16 mixture (solid line) and CrIDA3-CrODA16 complex (dotted line). Lower panel: SDS-PAGE analysis of SEC fractions of the CrIFT46/56+CrIDA3+CrODA16 mixture. Results show CrODA16 co-eluting either with CrIFT46/56 (peak 1) or with CrIDA3 (peak 2), with no evidence of a tetrameric complex formation.

**Figure S5: Characterization of CrArl3 and its interaction with CrODA16**

A) Size exclusion chromatography (SEC) profile of purified CrArl3_Q70L (upper panel). The corresponding SDS-PAGE analysis (lower panel) confirms the purity and expected molecular weight of the protein.

B) HPLC analysis of nucleotide content in purified CrArl3_Q70L. The chromatogram shows peaks for standard GDP and GTP solutions, while the CrArl3_Q70L sample (black line) shows minimal nucleotide content, indicating it is in an unloaded state.

C) AlphaFold 3 prediction of the CrArl3-CrODA16 complex. Left: 3D structure model with confidence scores indicated by color. Right: Pairwise aligned error (PAE) plot showing the predicted accuracy of residue pair distances in the model. Lower scores (darker green) indicate higher confidence in the predicted interactions.

D) Surface electrostatic potential of the CrODA16-Arl3 complex model, with CrIDA3 overlaid. CrODA16 is shown as a surface, CrArl3 as a cyan cartoon, and CrIDA3 as a green cartoon. The magnified inset highlights the interaction surface between CrODA16 and CrArl3, with red indicating negative charge and blue indicating positive charge. Notably, CrIDA3 and CrArl3 occupy overlapping binding sites on CrODA16, suggesting mutually exclusive binding to CrODA16.

**Figure S6: CrArl3 and CrIDA3 compete for binding to CrODA16**

A) Nickel pull-down assay with immobilized CrODA16-HT (his-tagged). SDS-PAGE analysis shows that both CrArl3 and CrIDA3 can bind individually to CrODA16-HT. When both proteins are present, they appear to compete for binding to CrODA16-HT.

B) Nickel pull-down assay with immobilized CrODA16-HT, testing different ratios of CrIDA3 to CrArl3 (1:3, 1:1, and 3:1). The SDS-PAGE results demonstrate that the relative amounts of CrIDA3 and CrArl3 bound to CrODA16-HT depend on their input ratios, further supporting competitive binding between CrIDA3 and CrArl3 for CrODA16.

**Table S1: AlphaPulldown results for human and *Chlamydomonas* ODA16 interactors**

This table presents the complete AlphaPulldown results for potential interactors of ODA16 in both human and *Chlamydomonas* systems. The table includes 1000 prey proteins for each organism, sorted in descending order based on the combined interface Predicted Template Modeling (ipTM) and Predicted Template Modeling (pTM) scores. These scores indicate the confidence of the structural prediction and relative positions of the subunits, with higher scores suggesting a higher likelihood of interaction. For human proteins, UniProt IDs are provided, while for *Chlamydomonas*, the gene IDs are listed.

Figure S1

**
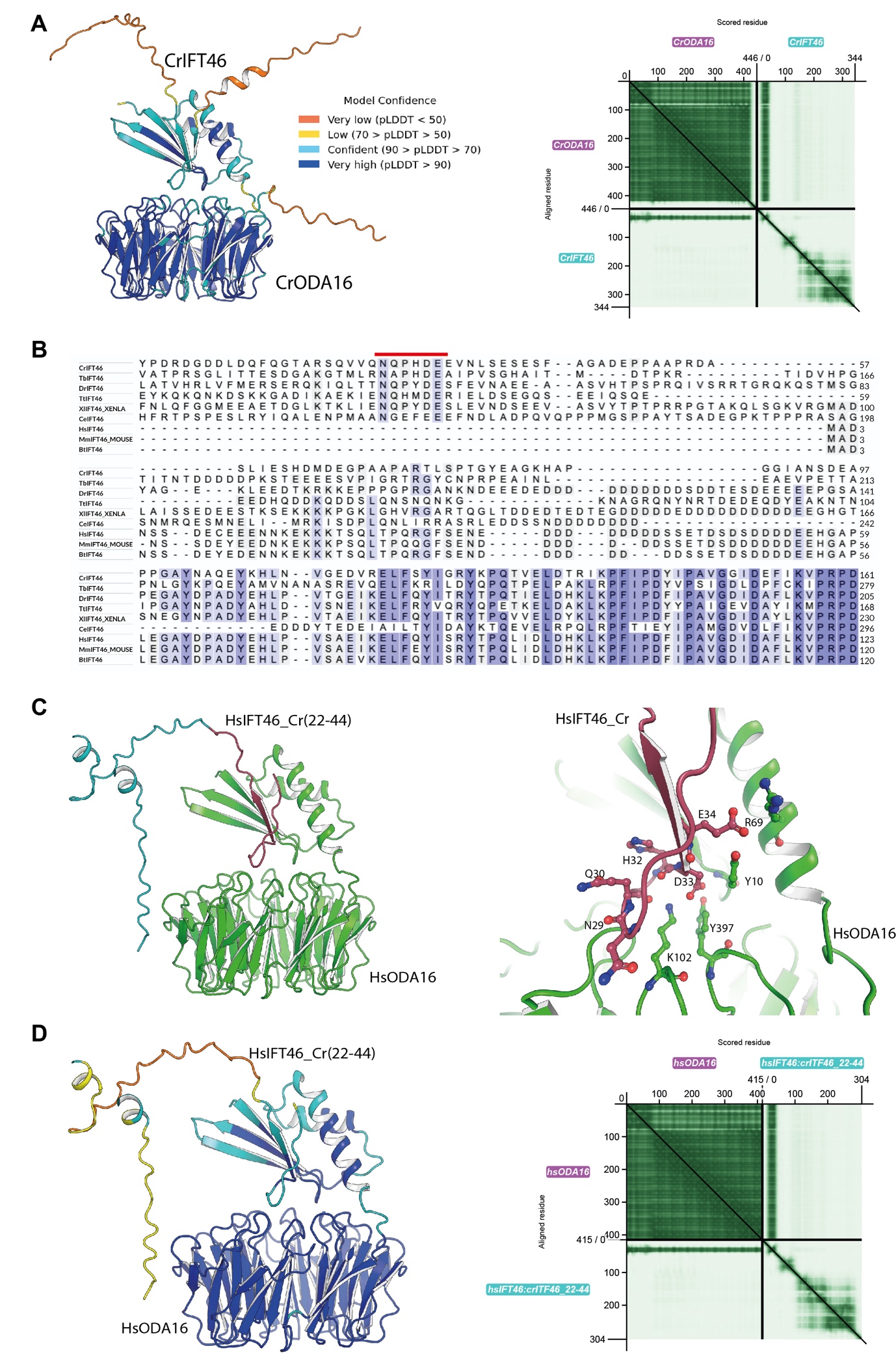
**

Figure S2


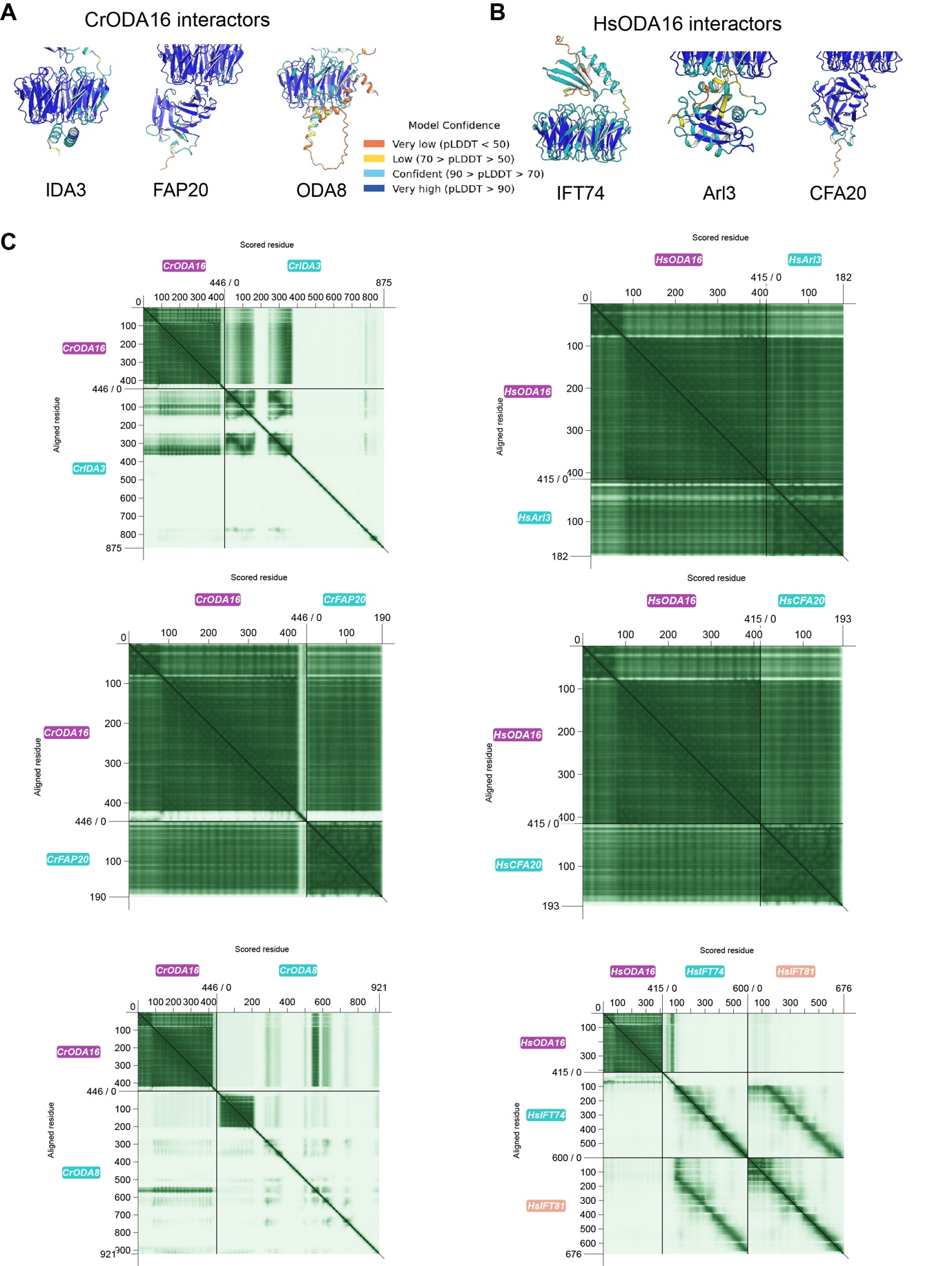


Figure S3

**
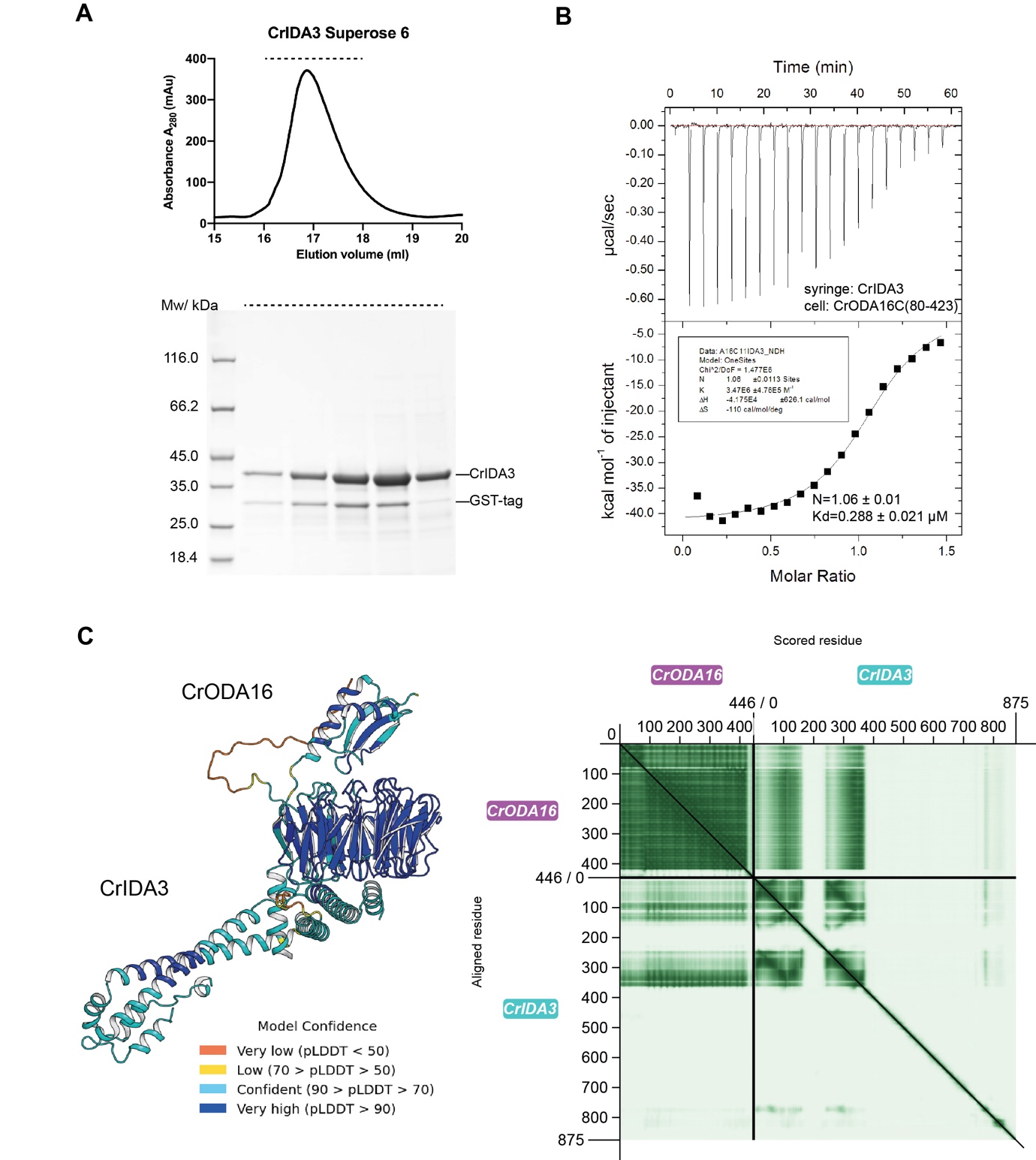
**

Figure S4

**
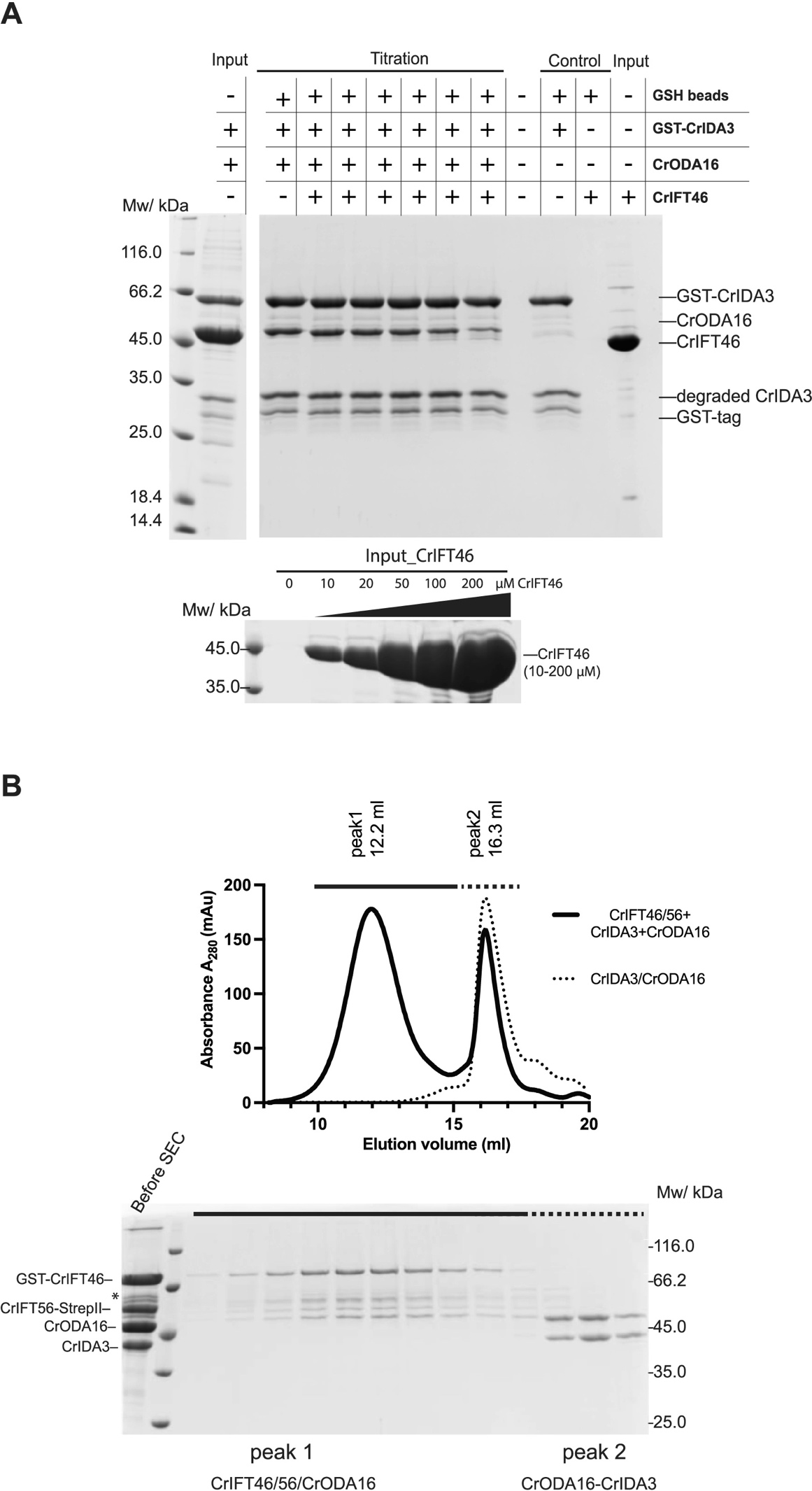
**

Figure S5


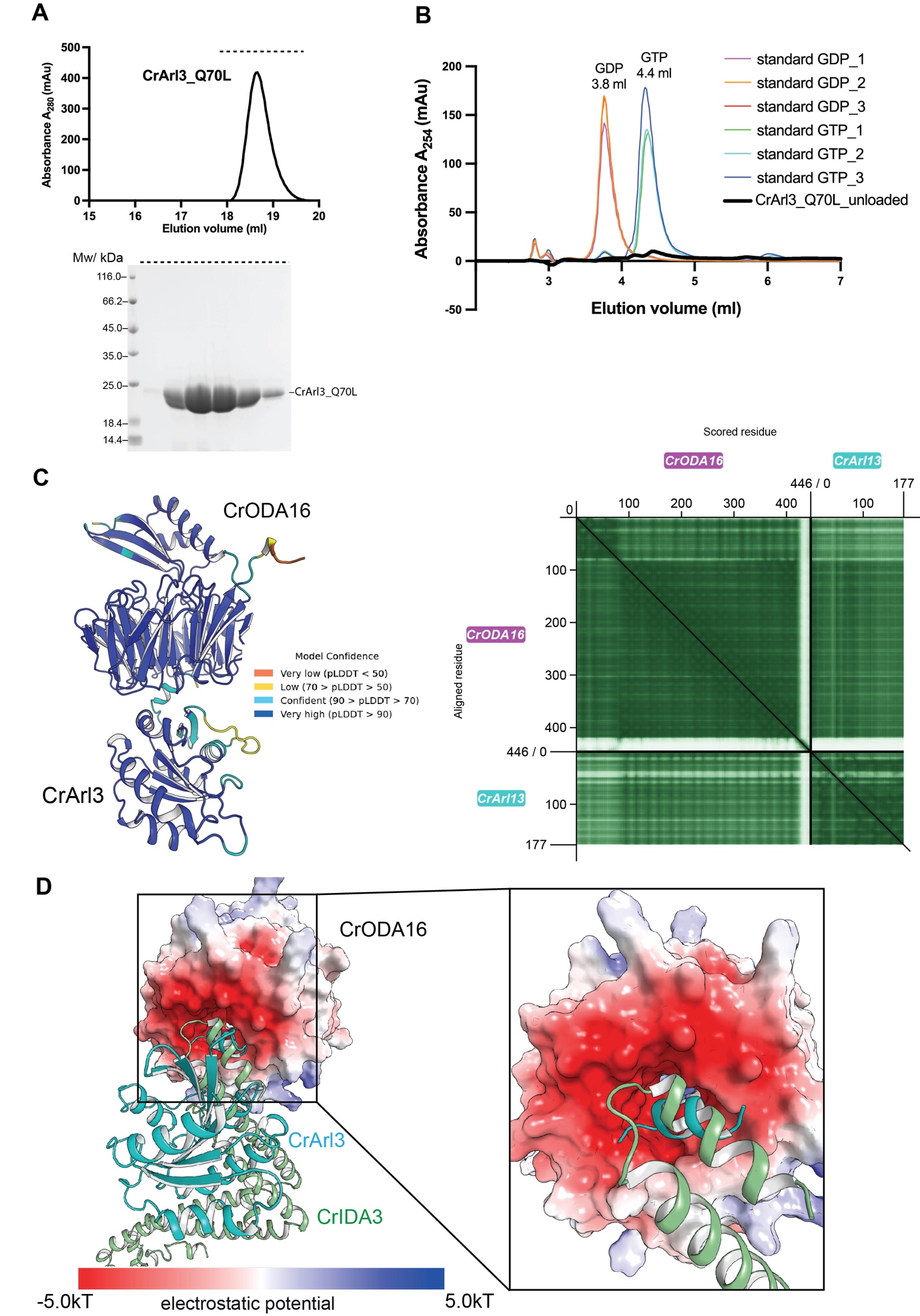


Figure S6

**
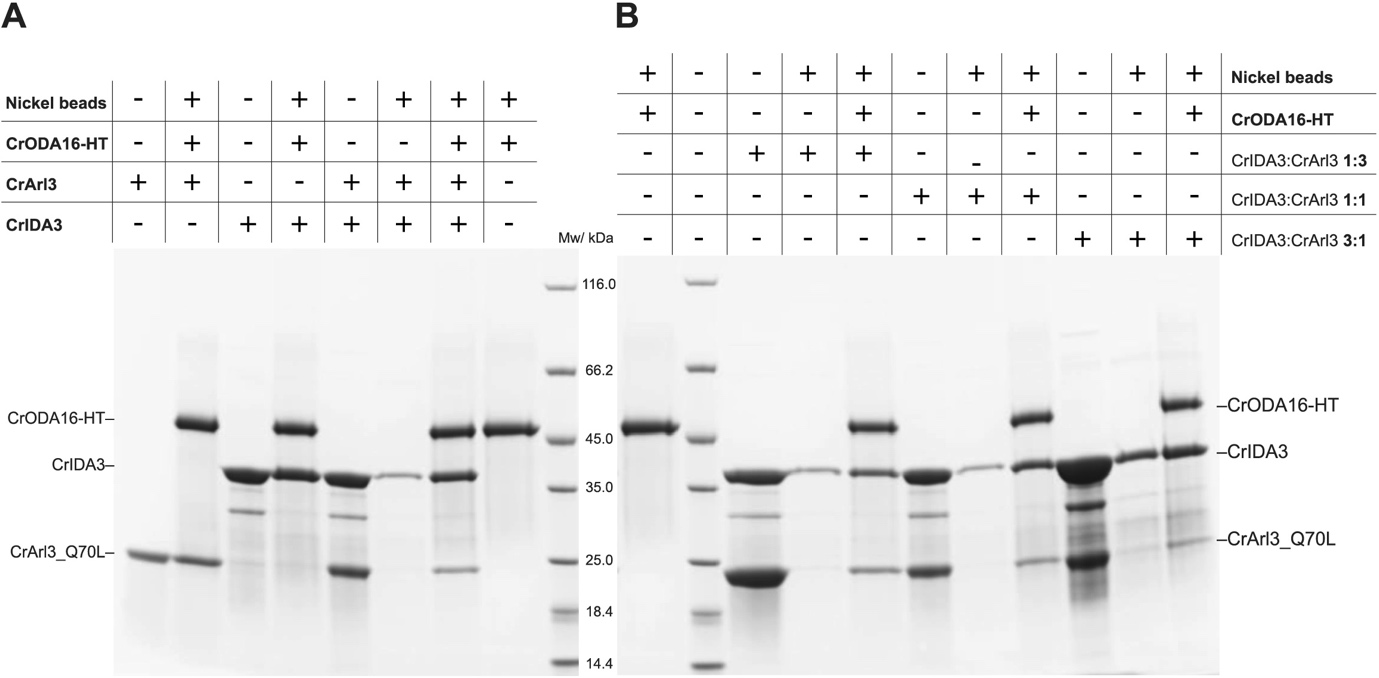
**
